# Global prevalence of domestic violence against adults during COVID-19 and its determinants: A systematic review, meta-analysis, and meta-regression analysis

**DOI:** 10.1177/17455057261465474

**Published:** 2026-07-02

**Authors:** Razieh Bidhendi-Yarandi, Farhad Nosrati Nejad, Akbar Biglarian, Payam Roshnfekr, Samira Behboudi-Gandevani

**Affiliations:** 1 Department of Biostatistics and Epidemiology, School of Social Health, 48533 University of Social Welfare and Rehabilitation Sciences, Tehran, Iran; 2 Social Determinants of Health Research Center, 48533 University of Social Welfare and Rehabilitation Sciences, Tehran, Iran; 3 Department of Social Welfare, School of Social Health, 48533 University of Social Welfare and Rehabilitation Sciences, Tehran, Iran; 4 Social Welfare Management Research Center, 48533 University of Social Welfare and Rehabilitation Sciences, Tehran, Iran; 5 Faculty of Nursing and Health Sciences, 1786 Nord University, Bodø, Norway

**Keywords:** COVID-19, domestic violence, systematic review and meta-analysis

## Abstract

**Background:**

Domestic violence (DV) is a pressing public health issue with worldwide implications. Crises like the COVID-19 pandemic can exacerbate this problem, as social and personal challenges intersect with the disruptive effects of health measures.

**Objective:**

This study aimed to determine the prevalence of DV during the COVID-19 pandemic and identify its associated determinants.

**Design:**

Systematic review and meta-analysis.

**Data sources and methods:**

A comprehensive review of the literature in PubMed/Medline, EMBASE, PsycINFO and Cochrane Covid-19 register up to July 2024 were conducted. Pooled prevalence of DV during COVID-19, including overall and subtype prevalence were assessed, while also extracting associated factors and determinants. Meta-regression analyzed the impact of determinants on heterogeneity. A random effects model estimated the pooled prevalence using the meta-prop method, applying Freeman-Tukey double arcsine transformation for variance stabilization.

**Results:**

A total of 37 qualifying studies were included in our final analysis with a total sample size of 160,701 individuals. The overall pooled prevalence of DV, regardless of subtypes, was 13% (95% CI: 12-14%). Among the different subtypes, psychological violence had the highest pooled prevalence of 24% (95% CI: 19–28%), while financial violence had the lowest pooled prevalence of 7% (95% CI: 6–9%). Sexual violence (11%, 95% CI: 9–12%), physical violence (10%, 95% CI: 9–12%), and violence involving threats and controlling behaviors 11% (95% CI: 8–13%) had approximately similar prevalence rates. Meta-regression analyses showed the risk of overall DV was significantly higher among younger women Risk Difference (RD) =15% (P<0.001), 40 years and younger individuals RD=12% (P<0.001), and those living in developing countries RD=14% (P<0.001) compared to their counterparts. On average, women had a higher risk of overall DV than the elderly and men RD=9% (P<0.001).

**Conclusion:**

The study revealed a concerning global prevalence of DV at 13% during the COVID-19 pandemic, with psychological violence being the most common subtype. Women and young adults were identified as particularly vulnerable, with risk differentials of 15% and 12%, respectively, notably higher in developing countries (14%). This comprehensive assessment of DV prevalence and determinants offers valuable insights for future research and public health strategies.

**Registration:**

PROSPERO CRD42022351634.

## Plain language summary

DV is a serious global problem that affects people’s safety, health, and well-being. During the COVID-19 pandemic, many families faced additional stress because of lockdowns, financial difficulties, and restricted access to support services. These challenges created conditions that could increase the risk of violence at home. In this study, we reviewed and combined results from 37 research papers from around the world to understand how common DV was during the pandemic. Altogether, these studies included more than 160,000 participants. We found that about 13 out of every 100 people experienced some form of DV during COVID-19. Emotional or psychological violence, such as insults, intimidation, or controlling behaviors, was the most common type. We also examined who was most at risk. Our findings show that women, especially younger women and adults 40 years and younger, faced a significantly higher risk of experiencing violence. Higher rates were also observed in developing countries, where economic instability, limited access to support services, weaker social protection systems, and cultural barriers to reporting violence may have increased both the risk of DV and the difficulty of seeking help during the pandemic. Understanding the scale and risk factors of DV during crises can help governments and organizations improve support services. Our findings highlight the need for accessible reporting systems, targeted protections for vulnerable groups, and stronger social support during emergencies.

## 1. Introduction

Domestic violence is a complex and multidimensional global public health concern encompassing various forms of physical, sexual, psychological, and financial abuse, as well as controlling and coercive behaviors.^1,2^ These acts of violence are often perpetrated by household members.^3^ Women are often the victims of DV, with approximately one in three women experiencing physical or sexual violence during their lifetime.^4,5^ Despite ongoing efforts to reduce DV, it remains a persistent problem worldwide, often exacerbated by factors such as natural disasters and socio-economic crises.^6,7^

The COVID-19 pandemic has significantly disrupted societal norms, leading to profound psychosocial and social consequences.^8^ Mandated lockdowns, quarantines, remote work, and required home isolation have altered household dynamics, leading to heightened stress, economic instability, and reduced access to support systems.^8–10^ These conditions have contributed to an alarming rise in DV incidents.^11^

However, results of studies from previous epidemics and natural disasters have produced mixed findings on their effects on DV.^12,13^ For example, during the 2014 Ebola outbreak in West and Central Africa and the 2017 cholera outbreak in Yemen, increased DV rates were reported, along with disruptions to family relationships, social welfare systems, and community protection services.^14^ Conversely, other research suggested that pandemics and disasters may reduce violent crime, enhance family cohesion, and promote prosocial behavior, possibly by reinforcing community trust and cooperation.^15^ However, results of a systematic review in the early stage of the pandemic showed that the incidence of DV rates increased post-lockdowns. Interestingly, the effect was more pronounced when only US studies were considered.^13^

The prevalence of DV during the COVID-19 pandemic has thus emerged as both a significant public health concern and a global human rights issue. Addressing this crisis requires the development of evidence-based strategies and recommendations to reduce DV during both short- and long-term catastrophic events.^16,17^

Various determinants have been identified as contributing factors to this rise. Economic stressors, such as job loss and financial instability, have heightened tensions within households, leading to increased conflict and violence. Social isolation, a common consequence of lockdown measures, has reduced access to support networks and resources, leaving victims more vulnerable to abuse. Additionally, the disruption of services, including shelters and counseling, has further impeded victims’ ability to seek help. Mental health issues, exacerbated by the pandemic, also play a crucial role, as increased anxiety and depression can lead to heightened aggression in potential perpetrators.^18–21^

Vulnerable groups such as women, children, and the elderly could be more susceptible to DV due to their social and economic dependencies, which often leave them without the means to escape abusive situations. These populations may face unique challenges, including isolation, lack of access to resources, and societal stigmas that discourage them from seeking help. Furthermore, the dynamics of power and control in abusive relationships can disproportionately affect these groups, making it essential to develop targeted interventions and support systems that address their specific needs and vulnerabilities. Understanding these determinants is essential for developing effective interventions and support systems to address the surge in DV during and after the pandemic.^22,23^

To contribute to this objective, this systematic review and meta-analysis aimed to investigate the prevalence of DV during the COVID-19 pandemic, as well as the associated determinants.

## 2. Methods

This study adhered to the Preferred Reporting Items for Systematic Reviews and Meta-analyses (PRISMA) reporting guidelines.^24^The protocol of this study was registered in PROSPERO (CRD42022351634). The project was found to be in accordance with the ethical principles of Research Ethics Committees of University of Social Welfare and Rehabilitation Sciences: IR.USWR.REC.1401.140.

The review question was framed based on the PI/ECO statement as follows: The population (P) included individuals affected by the COVID-19 pandemic, as defined by the studies, with no restriction based on gender or ethnicity. The exposure (I/E) was the COVID-19 pandemic as a period or context in which DV occurred. No specific comparison (C) group was included. The primary outcomes (O) were the pooled prevalence of DV during COVID-19 including overall prevalence and subtypes of physical, sexual, psychological, economic violence and controlling behavior violence. As secondary outcomes all factors and determinants associated with DV were extracted.

### 2.1. Eligibility criteria

Observational studies were considered eligible if they have a population-based design utilized multicenter data or data registries, reported the number of cases or prevalence of DV during the COVID-19 pandemic, and reported potential determinants. Studies focused on minor populations such as pregnant women, individuals with specific disorders, and children and adolescents under 18 years were excluded. DV includes any actions within a family or domestic setting that inflict physical, psychological, sexual, or economic harm on another individual, regardless of whether the victim and perpetrator are biologically or legally related. It can take place between current or former intimate partners and also encompasses abuse directed towards children, siblings, and the elderly. Studies with unclear definitions of DV, as well as those that relied on newspaper articles or Google data, were also excluded. Additionally, studies focusing on help-seeking centers data for DV, including referrals from police, hospitals, or physicians where the cases may only reflect more severe forms of violence were excluded. Child violence was excluded from this systematic review as it involves different risk factors, reporting mechanisms, etc. Compared to violence among adults. Studies published in languages other than English, those with unclear or missing data, case reports, conference proceedings, reviews, and letters were also not eligible for inclusion.

### 2.2. Search strategy

We conducted a comprehensive literature review using databases such as PubMed (including Medline), EMBASE, PsycINFO, and Cochrane Covid-19 register up to July 2024. Additionally, we also manually searched the reference lists of selected studies and other relevant reviews to maximize the identification of eligible studies and consider results from grey literature.

A combination of synonyms and relevant terms incorporated Medical Subject Headings (MeSH), and free text was employed to maximize the sensitivity of the search across electronic databases used to identify relevant studies and titles, abstracts, and keywords. The following keywords were used: (“Domestic Violence” OR “Spouse Abuse” OR “Elder Abuse” OR “Intimate Partner Violence” OR “intimate terrorism” OR “coercive controlling violence” OR “Physical Abuse” OR “verbal abuse” OR “sexual abuse” OR “Homicide” OR “Sexual assault” OR “physical assault” OR “verbal assault” OR “Family violence” OR “Intimate Partner Aggression” OR “interpersonal violence” OR “partner violence” OR “Domestic harassment”) AND (“Coronavirus” OR “COVID-19” OR “COVID19” OR “SARS-CoV-2” OR “2019 novel coronavirus” OR “2019-nCoV” OR “pandemia” OR “global pandemic” OR “lockdown”). To ensure optimal retrieval of relevant articles, we customized the search strategy for each database.

### 2.3. Study selection and data extraction

Titles, abstracts, and full texts of selected studies were independently screened by two authors based on the eligibility criteria. Disagreements or conflicts were resolved through discussion and consensus, resulting in complete agreement on the final set of included studies. In cases of missing data or unavailable full texts, the corresponding authors were contacted. If no response was received, the articles were excluded. The following data were extracted from eligible studies: the first author’s name; publication year; country, study design; sample size; characteristics of population, phase of COVID-19 Pandemic, types of DV and measurement tool, details of the abuser and DV prevalence along with its determinants. The accuracy of data before the meta-analysis, the data extraction process was double-checked to avoid bias in data entry and extraction. Any discrepancies were resolved through discussion with the senior review author.

### 2.4. Assessment of methodological quality

We used the JBI Critical Appraisal Checklist^25^ for Prevalence Studies, which includes criteria for evaluating aspects such as study design, sample selection, measurement methods, and statistical analysis. Two independent reviewers conducted the methodological quality assessment of each included study. Each reviewer assessed the studies separately to ensure a thorough evaluation. Any disagreements between the reviewers regarding the appraisal of study quality were resolved through discussion. If consensus cannot be reached, a third reviewer, who is not involved in the initial assessment, were consulted to adjudicate and provide a final decision. The results of the quality assessments were documented and used to inform the interpretation of the review’s findings. Studies were classified based on their methodological quality to help synthesize and analyze data.

### 2.5. Data analysis

The analysis was conducted in two phases. Initially, a systematic review of the determinants of DV during the COVID-19 pandemic was performed. This involved a comprehensive search including identifying key factors associated with DV. The identified factors were then categorized into distinct themes based on their similarities, allowing for a structured understanding of the various influences contributing to the prevalence of DV. The STATA software package (version 14; STATA Inc., College Station, TX, USA) and R software metaphor package version 4.4.1 were used for statistical analysis.^26^ Heterogeneity was evaluated using the I^2^ index. Publication bias was assessed using funnel plots and the Egger test. In case of significant results, the trim and fill approach was applied. We utilized meta-analysis to derive pooled prevalence estimates of DV across diverse populations, enabling us to gain a comprehensive understanding of its occurrence. To achieve this, we employed a random effects model, which accounts for variability between studies and provides a more accurate estimation of the overall prevalence. Specifically, we applied the meta-prop method to calculate these pooled estimates. To enhance the stability of the variances and improve the reliability of our results, we implemented the Freeman-Tukey double arcsine transformation. This transformation helps to mitigate issues related to proportion data, particularly when dealing with proportions close to 0 or 1, ensuring that our estimates reflect a more precise picture of DV prevalence across the examined studies.

Forest plots for each outcome and by any extracted subgroups such as gender (Male vs. Female based on the reported evidence), regions (Developed vs. Developing countries based on the world bank), type of DV (Physical, psychological/emotional/verbal, sexual, financial based on the reported evidence), age (40 years and younger vs. over 40 years based on the sensitivity analysis) were illustrated as well. Additionally, we conducted a subgroup analysis based on participants (Women, Men, Elderly), region (Developing vs. developing countries), age (40 years and younger vs. over 40), and phase of the COVID-19 pandemic categorized as follows:(i) Strict Lockdown (March 2020–June 2020), (ii) Partial Restrictions (July 2020–December 2021), and (iii) Post-Lockdown (January 2022 onwards). The strict lockdown period marked the initial global response to COVID-19, following the WHO’s declaration of a global health emergency on January 30, 2020, and a pandemic on March 11, 2020. During this phase, most countries implemented widespread stay-at-home orders, strict mobility limitations, and border closures to contain the virus.

Meta-regression analysis was conducted to evaluate how various extracted determinants contribute to the observed heterogeneity in the prevalence estimates of DV. By treating these determinants, such as socioeconomic factors, geographic location, and specific circumstances during the pandemic, as independent variables, we aimed to identify their influence on the dependent variable, which is the prevalence of DV. This analytical approach allows us to explore potential relationships and variations in outcomes across different studies, helping to pinpoint which factors may significantly impact the rates of DV. By understanding these relationships, we can better explain the variability among studies and enhance our insights into the underlying causes of DV during the examined period.

Sensitivity analysis using “*metaninf*” STATA package was conducted to identify the influential studies. This method systematically excludes each study one at a time and recalculate the overall effect size to observe how each study influences the results. Forest plots for each DV outcome were drawn to visually represent the influence of each study on the overall effect size. Correlation tests were performed to assess the relationships between independent variables to identify any potential multicollinearity issues as well.

## 3. Results

### 3.1. Search results and characteristics of the included studies

**Figure 1.**
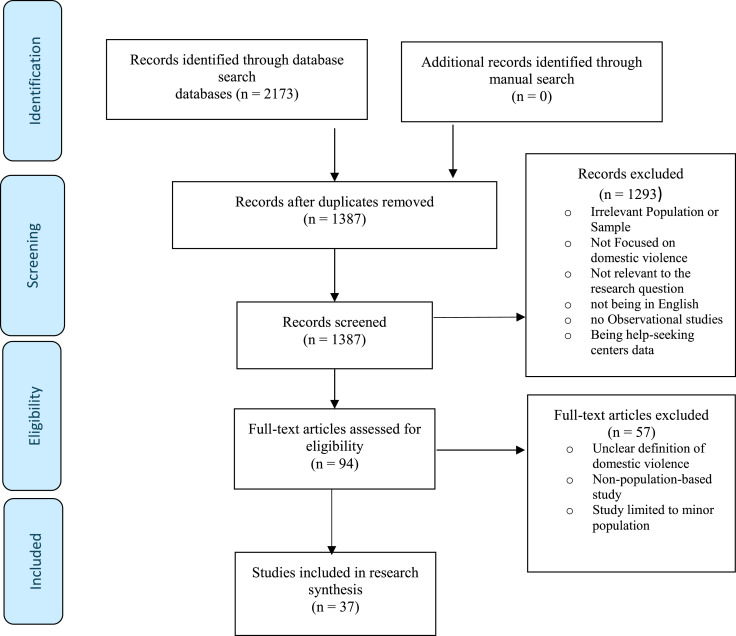
Flowchart of the search process.

A total of 2,173 records were initially retrieved from the database and hand-search strategies. After removing 786 duplicates, 1,387 unique records remained for title and abstract screening. Of these, 1,293 articles were excluded because they clearly did not meet the inclusion criteria (e.g., irrelevant outcomes, non-DV populations, non-COVID-19 context, and commentary/editorials). This left 94 full-text articles for detailed eligibility assessment. Following full-text review, 57 studies were excluded for reasons such as: not reporting DV prevalence, not assessing DV during the COVID-19 pandemic, inappropriate study design (e.g., qualitative-only), or lack of accessible full text. Ultimately, 37 studies met the inclusion criteria and were included in the quantitative synthesis. These steps are summarized in in Figure 1, the PRISMA flow diagram for clarity.^27–63^

No studies were excluded due to missing essential data or failure to obtain full texts, as all potentially eligible full-text articles were successfully retrieved. The main characteristics of the included studies are summarized in Table 1. A total of 11 studies collected data exclusively during the Strict Lockdown phase,^30,35,39,41,43,45,47,51,52,54,63^ 21 studies during the Partial Restrictions phase,^27–29,31,33,34,37,38,40,42,46,49,50,53,55–61^ and 4 studies during the Post-Lockdown period.^32,44,48,62^ However, one study did not specify the pandemic phase,^36^ and 8 studies collected data across both the Strict Lockdown and Partial Restrictions phases.^34,37,38,45,50,53,56,59^ These studies were categorized based on the majority of their data collection period. Number of records for each subgroups of diversity of participants, region, and age group for each Domestic violence type were reported in Table 2.

**Table 1.** Main characteristics of included studies.

Author, year	Country	Method of study	Sample	Sample size	Age (mean)	Phase of COVID-19 pandemic	Types of DV	Main research questionnaires	Factor associated with domestic violence
Agero G, et al. 2024^27^	Ethiopia	Community-based cross-sectional study	Reproductive-Aged Women,15-49 years old	1458	28 (IQR: 11)	15 to March 30, 2021	Physical, sexual, psychological, controlling behavior, economic violence	Structured questionnaire adapted from previous studies and WHO standard questions for the assessment of Violence Against Women (VAW).	**Economic Factors:** Lower monthly income **Behavioral Factors**: (Quarreling with partner’s family, witnessing childhood family violence, decisions made solely by husband or wife) **Partner Characteristics**: (Partner’s educational level (e.g., unable to read/write), drinking alcohol, aggressive behavior towards other men, partner’s family involvement in decision-making)
Arenas-Arroyo E, et al. 2021^28^	Spain	Cross-sectional study, using national online survey	Couples living together	8951	NM	After the lockdown, May 17th and June 12^th^ 2021	Reported and non-reported non-extreme violence, Physical, sexual Psychological violence	Ad-hoc Questionnaire	**Lockdown:** Increases in IPV are observed when both partners are locked down, with significant effects on psychological and sexual abuse but not on physical violence. **Economic Stress:** Increases in IPV are observed when either or both partners experience economic stress. The effects are significant across all types of IPV, with the largest effects on psychological abuse. **Pre-existing IPV:** Couples with previous exposure to IPV show a more pronounced increase in IPV compared to those without prior exposure.
Campbell et al. 2023^29^	Multiple country (including Low or low-middle, Upper-middle and High-income country)	Cross-sectional study Online survey	General population	23067 (for analysis: 15336)	^#^34.3 (12.8)	July 20th, 2020, to February, 15th, 2021	Physical, Sexual violence	Shortened version of the WHO IPV scale	**Sex:** Higher IPV risks associated with male at birth and non-binary identities, especially for sexual violence. **Sexual Orientation:** Increased risk for asexual and pansexual individuals, particularly during pandemic restrictions. **Economic Factors:** Economic vulnerability linked to higher IPV risks. **Cohabitation Status:** Higher IPV risks for those cohabiting with partners. **Geographic Factors:** Stringency of lockdown measures and urban versus rural residence impacted IPV rates.
Chang et al. 2021^30^	USA	Cross-sectional population-based study, using online survey	60 years and older adults	897	^#^68.5 (5.3)	April 23 and May 5, 2020	Financial, Verbal and Physical abuse	Adapted from the Hwalek-Sengstok Elder Abuse Screening Test (H-S/EAST) and the Vulnerability to Abuse Screening Scale	**Protective Factors:** • Greater sense of community • Higher adherence to physical distancing **Risk Factor:** • Higher pandemic-related financial strain
Chang, et al. 2024 1^31^	Multiple^24^ countries	Cross-sectional online survey (secondary analysis of the I-SHARE survey)	Adults older than 45 years	2867	NM	July 2020 to February 2021	Physical, sexual, psychological violence	WHO Violence Against Women Instrument (VAWI)	**Risk factor:** Social isolation, an increase in food insecurity, middle-aged and older adults **Protective factor:** stringency of social distancing measures
da Costa Siqueira et al. 2023^32^	Brazil	Cross-sectionalepidemiological study	Female aged 18 years or older	1086	NM	January and May 2022	Psychological/emotional, Physical, Sexual violence	World Health Organization Violence Against Women (WHO VAW STUDY)	Perceived stress
Demeke et al., 2023^33^	Ethiopia	A community-based cross-sectional study	Women of reproductive age 18-45 years	796	NM	2021	Psychological, physical/emotional, and sexual violence	WHO instrument on violence against women	**Woman-Related Factors**: • No formal education • Having no income • Acceptance of IPV **Male Partner-Related Factors**: • No formal education • Alcohol consumption • Smoking habits **Family-Related Factors**: • Family size Family size more than 4-5 members
Drieskens et al. 2022^34^	Belgium	Online Surveys	All population older than 18 years	April 2020 Survey: 25,251 March 2021 Survey: 12,589	NM	April 2020 and March 2021	verbal/psychological, physical, sexual violence	Ad-Hoc tool	• Being unsatisfied with social contacts • Perceived weak social support • Less confidence in health care services • Social loneliness • Emotional loneliness
Du et al., 2021^35^	China	Cross sectional survey	Elderly people over 65 years of age.	10362	NM	April to May 2020	physical, emotional, financial abuse and neglect	Elder abuse questionnaire from the “Third Survey on Chinese Women’s Social Status	• Age • Gender • Marital status • Education level • Income • Number of children • Health condition • Cognitive ability • Religious belief
Every-Palmer et al., 2020^36^	New Zealand	Cross sectional demographical Study	Adult 18 and over	2010	NM	NM	Physical assault, Harassment and threatening Behaviour, Sexual assault, Frightened by a family member	Ad-hoc questions designed specifically to measure family violence, suicidal ideation, and alcohol consumption	**Satisfaction with Household Members During Lockdown**: Dissatisfaction with household members was associated with higher psychological distress. **Health Vulnerabilities**: Those with health vulnerabilities reported higher psychological distress. **Self-Rated Health**: Poor or fair self-rated health was associated with higher psychological distress. **History of Mental Illness**: A past history of mental illness was associated with higher psychological distress. **Work Loss**: Participants who experienced work loss or reduced work reported higher psychological distress.
Ezenwoko et al., 2023^37^	Nigeria	Cross sectional national survey study	Adults older than 18 years in relationships for more than one year	538	^#^37.2 (8.0)	May 22nd- July 27 2020	**Physical Violence**: Partner pushed, punched, twisted arm, kicked, or attempted to choke/burn **Sexual Violence**: Sexual deprivation, forced sex, forced sexual acts **Emotional Violence**: Insulting, humiliating, threatening **Economic Violence**: Refusal to provide money for household expenses, taking earnings or savings	Ad-hoc questionnaire	• Being men, • Social Class, • living in urban area, • being married, • lack of supporting system, • Normalization of violence, • Privacy Concerns, • Lockdown Stress
Fereidooni et al. 2023^38^	Iran	Population-based cross- sectional survey	Iranian women aged 18 to 60 years	2116	37.4 years	First 6 months of the pandemic	Physical, Psychological Sexual violence, Any other types (including multiple types)	World Health Organization multicountry study questionnaire on women’s health and domestic violence	**Socioeconomic Status (SES)**: Lower SES was strongly associated with higher IPV risk. **Change in Woman’s Job Status**: Unemployment or remaining a housewife were associated with increased IPV. **Change in Spouse’s Job Status**: Reduced job status of the spouse (from full-time to part-time or unemployed) was strongly associated with higher IPV.
Gebrewahd et al., 2020^39^	Ethiopia	Community-based cross-sectional study	Reproductive age women	682	^#^29.78 (5.78)	April to May, 2020	Psychological, physical and sexual violence	WHO core questionnaire on domestic intimate partner violence	• Women’s Age • Women’s Uneducated • Being Housewife • Being married • Husband’s Aged 31–40 years • Higher Number of Children: • Lower Family Monthly Income • Husband’s Alcohol Consumption • History of Husband’s Aggressive Behavior:
Gharacheh et al. 2023^40^	Iran	Cross-sectional study	Married women attending healthcare centers	5317	^#^34.37 (9.66)	November 2020 to September 2021,	Physical, Psychological Sexual violence	Revised Conflict Tactics Scales (CTS2)	• Low levels of social support • Shorter duration of marriage • Unemployment of both women and their spouses • Poor economic status • Substance abuse by the husband including Alcohol use, Drug abuse, Smoking
Gilchrist et al., 2023^41^	13 countries	Survey	Partners with various genders	35854	^#^37.1 (13)	May -June in 2020	Financial control, Emotional abuse, Coercive control, Threatening behavior, Technology facilitated Abuse, physical Abuse, sexual abuse, any other	Ad-hoc tool	• Higher psychological distress, • Poor coping with pandemic-related changes, • Tension in relationship and its changes (increases or increases and decreases) • Alcohol consumption reported
Gleason et al. 2021^42^	USA	National survey	Men and women as Partners	1051	^#^38.54 (10.56)	October 2020	Physical and sexual violence	Domestic violence risk assessment questionnaire.	NM
Glowacz et al. 2020^43^	Belgium	Population based cross-sectional online survey	Adults engaged in a romantic relationship and living together	1532	^#^35.94 (14.84).	April 17 to 1 May 2020	Physical assault, psychological aggression	Ad-hoc tool (self-designed questionnaire)	• Suffering from Anxiety and depression • Conditions of lockdown, such as time spent at home, and living environment
Arumugaperumal et al. 2024^44^	India	Community-based cross-sectional study	Women ever married or partnered of age ≥18 years	190	34 (8) years	June 2023 to August 2023	Controlling behavior, physical and sexual violence	WHO Violence Against Women Instrument	Suffering from depression
Indu et al. 2021^45^	India	Community-based cross-sectional survey	Married women 18-45 years	209	^#^36.21 (8.05)	July- June 2020	Physical, psychological, and sexual violence	Domestic Violence Questionnaire (DVQ)	Suffering from depression and anxiety
Kliem et al. 2023^46^	Germany	Representative Population Surveys	Participants living with a romantic partner and parents with at least one child	3127 **(Survey 2021a**: 1383 **Survey 2021b**: 1744)	NM	Feb-March 2021, June-Oct 2021	-Physical violence in intimate partnerships -Sexualized violence in intimate partnerships	Family Maltreatment Measure (FM)	NM
Koga et al. 2022^47^	Japan	Population based survey study	Older adults (age ≥65 years)	18263	NM	April to May 2020	Physical, psychological, and financial elderly abuse	Self-administeredquestionnaire	Refrainment from daily activities (e.g., shopping, interacting with neighbors)
Leite et al. 2023^48^	Brazil	Cross-sectional, population-based study	Women aged 18 years and over who had or have had an intimate partner in the past 24 months	1086	NM	January to May 2022,	Psychological, physical, and sexual Violence	World Health Organization (WHO) instrument on violence against women	• Sociodemographic factors (e.g., less schooling, marital status, being single), • behavioral factors (e.g., alcohol use), • Family and life experiences (e.g., maternal aggression by an intimate partner, childhood sexual abuse)
Miall et al. 2023^49^	30 countries (including high-income, middle-income, and low-income countries)	Cross-sectional online survey	Cis-women 18–97 years	9004	32 years	July 2020 and February 2021	Psychological, physical, and sexual Violence, restrict social contacts (including online or telephone contact).	WHO instrument that measure physical, psychological and sexual IPV	Working from home during the pandemic work disruptions (e.g., unemployment, working reduced hours)
Miller et al. 2022^50^	Uganda	Population phone-based survey	Women aged 13–80 years	556	^#^33.4 (13,7)	June-August of 2020	Physical, verbal, sexual violence	10 questions adapted from the Conflict Tactics Scales (CTS)	• Protective factor: Higher general self-efficacy • Risk factor: Alcohol use
Morgan et al. 2020^51^	Australia	Population based online survey	women aged 18 years and over in cohabiting relationships	7514	NM	May 2020	physical or sexual violence	Self-administered tool	**Social Isolation**: Less frequent contact with family and friends outside the household was associated with higher likelihood of violence. **Time Spent at Home**: Spending less time at home was associated with a higher likelihood of experiencing violence, possibly as a response to prior violence. **Financial Stress**: Increase in financial stress during the pandemic was linked to a higher probability of first-time violence. Higher financial stress levels prior to the pandemic were also associated with a higher prevalence of violence. **Prior Experience of Violence**: Women with a history of violence were more likely to experience repeat violence during the pandemic. Women without prior violence had a higher likelihood of experiencing violence for the first time, particularly with increased financial stress and social isolation.
O'Hara et al. 2022^52^	Singapore	Cross-sectional online survey	Participants having a steady partner	259	29 years	April 2020.	Restriction of contact with others, verbal abuse, restriction of access to finances, physical violence, pressured sex and forced sex,	WHO IPV scale	**Before and During Lockdown:** Younger age, non-heterosexual orientation, and having more children in the household **During Lockdown Only:** Being of Chinese ethnicity and income moderate to upper-middle class
Plášilová et al. 2021^53^	Czech Republic	National online survey	Women older that 18 years old who living with a partner for at least 3 months before the onset of the pandemic	429	^#^48.45 (16.25)	12 March 2020 to 17 May 2020, 5 October to 25 November 2020	Economic, social, emotional, physical, sexual–psychological, sexual–physical	WHO IPV interview	Tension in the relationship with the partner, depression, partner support
Sanz-Barbero et al. 2021^54^	Spain	Cross-sectional study	Young men and women age 18–35	2515	NM	March–June 2020	Sexual violence (SV) including both intimate partner sexual violence and non-partner sexual violence	NM	**Sex:** Women were more likely to experience SV than men before the lockdown. **Sexual Attraction:** Higher prevalence of SV among individuals with bisexual or homosexual attraction. **Place of Birth:** Higher prevalence among those born outside Spain, though the significance varied across periods.
Sheridan-Johnson et al. 2024^55^	USA	Population based cross sectional study	Young adults ages 18 to 35	2112	^#^27.49 (0.16)	November 2020 and May 2021	Physical, sexual, or emotional aggression	Ad-hoc questionnaire	NM
Shewangzaw Engda et al. 2022^56^	Ethiopia	Community-based cross-sectional survey	Reproductiv aged women	700	^#^33.04 (7.5)	1 May to 1 July 2020	Emotional, sexual and physical violence	Women abuse screening tool (WAST)	• Depressive symptoms, • Overweight, • Suicidal ideation, • Body image disturbance
Shitu et al. 2021^57^	Ethiopia	Community based cross-section study	Reproductiv aged women	462	^#^26.5 (4.07)	September 20 to October 10/2020	Emotional, sexual physical violence	Ad-hoc questionnaire	• age ≥ 35, • Rural residence, • Husband’s educational status of lower than diploma, • Low social support
Son et al. 2022^58^	South Korea	Secondary data analysis of national data	Community-dwelling elderly, 65 years and older	3106	NM	July 21 to October 23, 2020	Physical abuse, psychological abuse, Financial exploitation, Neglect, Sexual abuse,	Ad-hoc questionnaire	**Risk Factors**: • Social isolation, • depressive symptoms, • physical dependency, • cognitive impairment, • number of comorbidities, • smoking, alcohol consumption (in this specific context), • possibly a greater number of family members. **Protective Factors**: • Higher recognition of abuse
Tadesse et al. 2022^59^	Ethiopia	Community-based cross-sectional study	Married or cohabited women	617 (total sample), 589 used in analysis	32 (IQR: 13)	26, 2020, to July 10, 2020	Physical, Emotional, Sexual	Adapted from Ethiopian studies, EDHS-2016, and WHO-2005 violence assessment tools	• Illiteracy of the woman • Illiteracy of the husband • Substance use by the husband (alcohol, chat, cigarettes) • Community tolerance attitude to violence
Yan et al. 2022^60^	Hong Kong	Community-based repeated cross-sectional survey	Community-dwelling older women aged 55 years and above	Wave 1: 910 Wave 2: 588	NM	December 2020 - January 2021	Physical abuse, psychological abuse, financial abuse	Three items capturing physical, psychological, and financial abuse	poorer self-perceived physical and mental health
Yan et al. 2022^61^	Hong Kong	Community-based repeated cross-sectional survey conducted via telephone interviews	Community-dwelling older adults aged 60 years and above	Wave 1: 1209 Wave 2: 819	NM	December 2020 - January 2021	Physical, Psychological (Verbal) and Financial Abuse	Ad hpc questionnaire	• Younger age, • Poorer subjective well-being, • Lower resilience
Yang et al. 2024^62^	China	Population based Cross-sectional online survey	Married or cohabitating population	2026 (1,058 men and 968 women)	Men: 38 (IQR: 33–47); women: 34 (IQR: 30–39.5)	April 29 to June 1, 2022	Physical IPV Sexual IPV Verbal IPV (insult, scream)	Adapted version of the Extended-Hurt, Insult, Threaten, Scream (E-HITS) scale	**For Women:** • Economic stressors including income loss, job loss, financial worry, • Household burden including presence of children, insufficient household supplies (food, water, daily supplies) **For Men:** • Economic stressors including job loss • Household burden: including presence of two or more children
Yari et al. 2021^63^	Iran	A national cross sectional survey	Adult women	203	38.59	May–June 2020	Physical, Emotional, and Sexual violence	Domestic violence questionnaire (17 items covering physical, emotional, and sexual violence)	Lower age (women aged ≤25 years), Illiteracy or primary education level, Previous marriage(s) Unwanted or unwise marriage

Violence against women (VAW), DV: Domestic Violence, Intimate partner violence (IPV), NM: Not Mention.

^#^mean (SD).

**Table 2.** Results of random effect meta-analysis of different types of domestic violence and subgroups of Diversity of participants, region, and age group.

Domestic violence type	Subgroups	^#^N	^@^Pooled prevalence (95%CI)	I^^^2	Egger’s publication bias test
Physical violence	Women	20	0.15 (0.09, 0.21)	99.27%	0.840
	Men	4	0.08 (0.06, 0.11)	72.97%	^$^--
	Elderly	6	0.03 (0.01, 0.04)	96.97%	0.436
	Developing countries	12	0.19 (0.10, 0.29)	99.50%	0.443
	Developed countries	26	0.05 (0.04, 0.07)	99.80%	0.059
	40 years and younger	22	0.13 (0.09, 0.18)	99.81%	0.234
	Over 40 years	5	0.06 (0.05, 0.07)	0.00%	0.400
	Strict lockdown	9	0.10 (0.07, 0.12)	95.85%	0.675
	Partial lockdown	21	0.09 (0.06, 0.12)	99.58%	0.876
	Post lockdown	3	0.13 (0.06, 0.19)	--	--
	Total	38	0.10 (0.09, 0.12)	99.45%	0.072
Psychological/emotional/verbal violence	Women	18	0.28 (0.18, 0.38)	99.55%	0.215
	Men	3	0.25 (0.23, 0.27)	--	--
	Elderly	6	0.13 (0.08, 0.17)	98.65%	0.435
	Developing countries	12	0.32 (0.20, 0.45)	99.60%	0.071
	Developed countries	20	0.18 (0.14, 0.22)	99.40%	0.090
	40 years and younger	19	0.28 (0.22, 0.35)	99.67%	0.657
	Over 40 years	3	0.10 (0.09, 0.11)	0.00%	^$^--
	Strict lockdown	9	0.22 (0.16, 0.27)	96.97%	0.843
	Partial lockdown	17	0.25 (0.15, 0.35)	99.50%	0.359
	Post lockdown	2	0.27 (0.25, 0.29)	--	--
	Total	32	0.24 (0.19, 0.28)	99.76%	0.660
Sexual violence	Women	20	0.12 (0.09, 0.16)	98.71%	0.995
	Men	4	0.10 (0.04, 0.16)	--	--
	Elderly	--	--	--	--
	Developing countries	12	0.18 (0.13, 0.24)	99.80%	0.122
	Developed countries	19	0.05 (0.04, 0.07)	98.45%	0.257
	40 years and younger	18	0.12 (0.08, 0.17)	99.60%	0.375
	Over 40 years	4	0.04 (0.01, 0.09)	96.47%	--
	Strict lockdown	7	0.09 (0.07, 0.11)	99.18%	0.342
	Partial lockdown	16	0.13 (0.10, 0.16)	98.48%	0.659
	Post lockdown	3	0.05 (0.04, 0.06)	--	--
	Total	30	0.11 (0.09, 0.12)	99.07%	0.209
Financial violence	Women	4	0.13 (0.08, 0.19)	94.46%	--
	Men	1	0.10 (0.08, 0.13)	--	--
	Elderly	6	0.03 (0.01, 0.06)	98.24%	0.500
	Developing countries	3	0.14 (0.08, 0.21)	^$^--	--
	Developed countries	10	0.05 (0.03, 0.08)	99.18%	0.152
	40 years and younger	5	0.11 (0.06 0.18)	98.48%	0.219
	Over 40 years	3	0.09 (0.07, 0.11)	0.00%	--
	Strict lockdown	3	0.08 (0.03, 0.13)	98.48%	0.511
	Partial lockdown	8	0.06 (0.04, 0.08)	0.00%	--
	Post lockdown	0	--	--	--
	Total	13	0.07 (0.06, 0.09)	98.30%	0.223
Others^*^	Women	6	0.20 (0.12, 0.28)	99.05%	0.325
	Men	0	--	--	--
	Elderly	30	0.08 (0.07, 0.09)	99.56%	0.500
	Developing countries	1	0.25 (0.23, 0.27)	--	--
	Developed countries	11	0.08 (0.03, 0.16)	99.83%	0.050
	40 years and younger	7	0.16 (0.05, 0.32)	99.86%	0.052
	Over 40 years	2	0.04 (0.03, 0.06)	--	--
	Strict lockdown	1	0.17 (0.13, 0.22	--	--
	Partial lockdown	7	0.06 (0.03, 0.09)	99.03%	0.534
	Post lockdown	1	0.57 (0.50, 0.64)	--	--
	Total	13	0.11 (0.08, 0.13)	99.20%	0.500
Overall	Women	71	0.18 (0.15, 0.21)	99.65%	0.851
	Men	13	0.11 (0.06, 0.17)	97.42%	0.404
	Elderly	21	0.06 (0.05, 0.07)	98.84%	0.844
	Developing countries	41	0.23 (0.17, 0.28)	99.55%	0.125
	Developed countries	94	0.08 (0.06, 0.10)	99.79%	0.050
	40 years and younger	77	0.17 (0.14, 0.20)	99.81%	0.050
	Over 40 years	17	0.06 (0.05, 0.08)	91.51%	0.972
	Strict lockdown	31	0.13 (0.11, 0.14)	99.68%	0.525
	Partial lockdown	75	0.13 (0.11, 0.15)	99.40%	0.650
	Post lockdown	9	0.19 (0.12, 0.25)	98.79%	0.050
	Total	134	0.13 (0.12, 0.14)	99.61%	0.250

^@^Obtained from Freeman-Tukey double arcsine transformation for proportion.

^#^Number of records.

^$^insufficient data.

^*^Include controlling or threatening behavior violence.

### 3.2. Quality appraisal, publication bias, and heterogeneity

The result of the quality of the study (risk of bias) is summarized in supplementary Table 1. All the studies have clearly defined their objectives, study settings, study subjects, inclusion criteria, measurement methods, statistical analysis, and outcomes. Based on the funnel plot and Egger’s test (supplementary Figures 2A-O), no statistically significant publication bias was detected for outcomes. However, the heterogeneity tests for all outcomes were significant (I^^2^> 80%); therefore, all estimations were obtained using the random effects method.

### 3.3. Results of meta-analysis

#### 3.3.1. Total results of different types of DV

Table 2 summarizes the pooled prevalence of overall DV and its different subtypes across different populations. The overall pooled prevalence of DV, regardless of subtypes, was 13% (95% CI: 12-14%). Among the different subtypes, psychological violence had the highest pooled prevalence of 24% (95% CI: 19–28%), while financial violence had the lowest pooled prevalence of 7% (95% CI: 6–9%). Sexual violence (11%, 95% CI: 9–12%), physical violence (10%, 95% CI: 9–12%), and violence involving threats and controlling behaviors (11%, 95% CI: 8–13%) had approximately similar prevalence rates. The forest plots (Supplementary Figures 1A–1D) visually demonstrate the wide variability in prevalence estimates across studies and highlight the consistency in the ranking of DV subtypes, with psychological violence consistently appearing as the most common form across settings. The subgroup forest plots further illustrate the differences across demographic groups, showing visibly higher pooled proportions among women and younger participants compared with men and older adults.

#### 3.3.2. Subgroup analyses based on participants, region, age and phase of COVID-19 pandemic

To improve clarity and avoid overlap among determinants of DV, we refined and consolidated categories into four distinct groups: economic stressors, lockdown-related restrictions, psychosocial stressors, and contextual or demographic risk factors. Economic stressors refer to job loss, income reduction, and financial insecurity. Lockdown-related stressors capture mobility restrictions, home confinement, and reduced access to services. Psychosocial stressors include increased anxiety, depression, substance misuse, and relationship conflict. Contextual/demographic factors include gender, age, household structure, and prior history of violence. Although economic stress was intensified by lockdown policies in many settings, we clearly separated these determinants based on their primary mechanisms: economic stressors reflect material or financial hardship, whereas lockdown-related stressors pertain to policy-imposed behavioral restrictions. The revised categorization facilitates a more accurate interpretation of how COVID-19 conditions influenced DV risk (Supplementary Table 2).

##### 3.3.2.1. Women

Table 2 provides the results of the random effect meta-analysis of different types of DV based on the various subgroups. Firstly, we performed a subgroup analysis among participants. The pooled prevalence of DV among women generally aligned with the overall trends observed in the total population but was notably higher than that of men and older adults (Table 2). The overall pooled prevalence of DV among women was 18% (95% CI: 15–21%). Among the different subtypes of DV, psychological violence had the highest pooled prevalence at 28% (95% CI: 18–38%), while financial violence had the lowest prevalence at 13% (95% CI: 8–19%). The pooled prevalence of controlling or threatening behavior violence was 20% (95% CI: 12–28%), followed by physical violence at 15% (95% CI: 9–21%) and sexual violence at 12% (95% CI: 9–16%) (Table 2).

##### 3.3.2.2. Men

The overall pooled prevalence of DV among men was 11% (95% CI: 6–17%). Among the different subtypes of DV, psychological violence had the highest pooled prevalence at 25% (95% CI: 23–27%), while financial violence was reported at a lower prevalence of 10% (95% CI: 8–13%). The pooled prevalence of sexual violence was 10% (95% CI: 4–16%), followed by physical violence at 8% (95% CI: 6–11%). The data for controlling or threatening behavior violence was insufficient for analysis (Table 2).

##### 3.3.2.3. Older adults

The pooled prevalence of DV among older adults was consistently lower than that of both women and men (Table 2). The overall pooled prevalence of DV among the elderly was 6% (95% CI: 5–7%). Among the different subtypes of DV, psychological violence had the highest pooled prevalence at 13% (95% CI: 8–17%), while financial violence was the lowest at 3% (95% CI: 1–6%). The pooled prevalence of physical violence was reported at 3% (95% CI: 1–4%), and the prevalence of sexual violence was not available due to insufficient data. Controlling or threatening behavior violence among older adults had a pooled prevalence of 8% (95% CI: 7–9%) (Table 2).

##### 3.3.2.4. Region of study (developing vs. developed countries)

Then, we performed a subgroup analysis based on the region of study. The overall pooled prevalence of DV was 23% (95% CI: 17–28%) in developing countries, whereas it was 8% (95% CI: 6–10%) in developed countries. The prevalence of different types of violence was as follows: physical violence was estimated at 19% (95% CI: 10–29%) in developing countries versus 5% (95% CI: 4–7%) in developed countries; sexual violence at 18% (95% CI: 13–24%) versus 5% (95% CI: 4–7%); psychological/emotional/verbal violence at 32% (95% CI: 20–45%) versus 18% (95% CI: 14–22%); financial violence at 14% (95% CI: 8–21%) versus 5% (95% CI: 3–8%); and other types, including controlling or threatening behavior, at 25% (95% CI: 23–27%) versus 8% (95% CI: 3–16%).

##### 3.3.2.5. Age group (40 years and younger vs. over 40)

Additionally, we performed a subgroup analysis based on age. The overall pooled prevalence of DV against the younger participants aged 40 years and younger was 17% (95% CI: 14-20%) compared to 6% (95% CI: 5-8%) among those aged over 40. The prevalence of different types of violence was as follows: physical violence was 13% (95% CI: 9–18%) in younger participants versus 6% (95% CI: 5–7%) in older participants; sexual violence was 12% (95% CI: 8–17%) versus 4% (95% CI: 1–9%); psychological, verbal, and emotional violence was 28% (95% CI: 22–35%) versus 10% (95% CI: 9–11%); financial violence was 11% (95% CI: 6–18%) versus 9% (95% CI: 7–11%); and violence for other reasons, such as threats and control, was 16% (95% CI: 5–32%) compared to 4% (95% CI: 3–6%) in younger versus older participants, respectively.

##### 3.3.2.6. Phase of the COVID-19 pandemic (strict, partial and post lockdown)

Finally, we performed a subgroup analysis based on the phase of the COVID-19 pandemic. The overall pooled prevalence of DV was similar during strict lockdown 13% (95% CI: 11%–14%) and partial lockdown 13% (95% CI: 11%–15%), but it increased post-lockdown 19% (95% CI: 12%–25%). The pooled prevalence of physical violence was 10% (95% CI: 7%–12%) during strict lockdown and 9% (95% CI: 6%–12%) during partial lockdown, with a slight increase post-lockdown 13% (95% CI: 6%–19%). For psychological violence, the pooled prevalence was 22% (95% CI: 16%–27%) during strict lockdown, 25% (95% CI: 15%–35%) during partial lockdown, and 27% (95% CI: 25%–29%) post-lockdown. The pooled prevalence of sexual violence increased from 9% (95% CI: 7%–11%) during strict lockdown to 13% (95% CI: 10%–16%) during partial lockdown, followed by a decrease post-lockdown 5% (95% CI: 4%–6%). Financial violence had a slightly higher pooled prevalence during strict lockdown 8% (95% CI: 3%–13%) compared to partial lockdown 6% (95% CI: 4%–8%). No studies assessed financial violence post-lockdown. The pooled prevalence of other forms of DV was 6% (95% CI: 3%–9%) during partial lockdown, while the highest prevalence was observed post-lockdown 57% (95% CI: 50%–64%), though this was based on limited number of data (Table 2).

### 3.4. Results of meta-regression, sensitivity analysis, and multicollinearity

**Figure 2.**
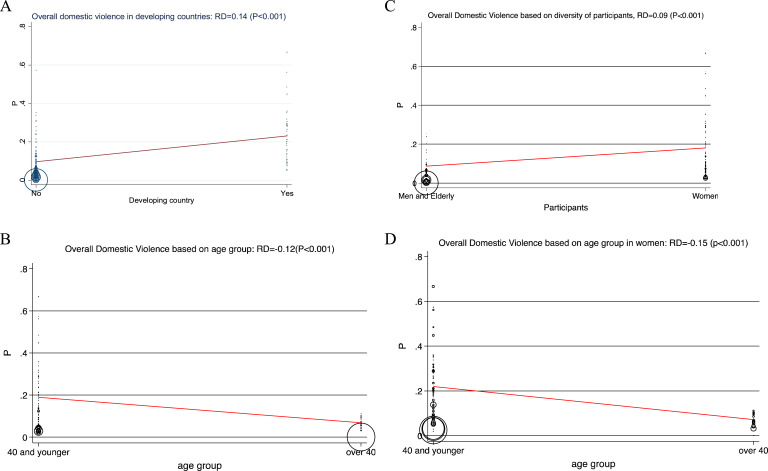
(A-D): Bubble plots of domestic violence to investigate sources of heterogeneity. RD=Risk Difference.

Results of the meta-regression showed that the risk of DV in developing countries was Risk difference (RD):14% higher (P <0.001) than in developed countries. Individuals 40 years and younger had a 12% higher risk of experiencing DV (P <0.001). On average, women had a higher risk of facing DV that was 9% higher (P < 0.001) than that of other participants, including the elderly and men. Younger women had a 15% higher risk of experiencing DV compared to those aged over 40 (P < 0.001). The bubble plots (Figure 2(a)–(d)) accompanying the meta-regression analyses provide a graphical depiction of the relationship between DV prevalence and key determinants, including age, gender, and country development level. These plots clearly show the upward trend in DV prevalence in women and in studies from developing countries and also downward trending line for older group population, reinforcing the statistical significance of these predictors in the meta-regression models.

#### 3.4.1. Systematic review results regarding determinants of DV

The review identified a range of factors associated with DV during the COVID-19 pandemic. These factors were categorized into seven key themes:

(i) Sociodemographic and Economic Determinants

Sociodemographic and Economic factors emerged as a significant driver of DV during the pandemic, 15 (41%) studies reported that economic vulnerability, lower monthly income, financial stress, job loss, especially when both partners were affected, working from home, being housewife, and work disruptions contributed to rising DV rates.^27–31,33,35,36,38–40,49,51,52,62^ Additionally, 14 (38%) studies^29,31,33,35,37,39,48,52,54,57–60,63^ indicated that age, educational levels, larger family size, and rural residency were linked to increased rates of DV.

(ii) Partners Relationship and Behavioral Factors

Fifteen (41%) studies revealed the dynamics of relationships and individual behaviors which played an important role in DV risk.^27,29,33,35–37,39–41,48,50,53,58,59,63^ Marital status, shorter duration of marriage, and previous marriage, particularly unwanted or unwise marriages, were linked to increased DV risk. Behavioral factors such as alcohol consumption, substance abuse, aggressive and controlling behavior, and decision-making autonomy within the relationship were also associated with DV. Additionally, misbehavior from the spouse’s family, conflict with in-laws, and family involvement in decision-making contributed to increasing the risk of violence.

(iii) Pandemic and Lockdown-Related Stressors

The COVID-19 pandemic introduced unique stressors that exacerbated the prevalence of DV.^28,31,34,37,41,43,47,49,51^ Factors such as prolonged time at home, privacy concerns, the inability to engage in daily activities like shopping or socializing with others especially neighbors, and the stress of working from home all contributed to increase DV.

(iv) Emotional, Mental, and Physical Health Vulnerabilities

Emotional, mental, and physical health conditions emerged as a significant factor influencing DV risk.^6,32,34–36,41,43–45,53,56,58,60,61^ Individuals experiencing emotional loneliness, low resilience, and psychological distress, including anxiety, depression, and cognitive impairment, were more likely to encounter DV. Additionally, those with physical health vulnerabilities, such as chronic health conditions, obesity, or physical dependency, particularly among older adults, may also increase the DV risk.

(v) Sexual Orientation and Gender Identity Factors

Six (16%) studies suggested that sexual orientations and gender identities were associated with DV.^29,30,35,37,52,54^ Individuals identified as non-binary, bisexual, or homosexual experienced increased risks of DV during the pandemic. While four studies reported higher rates of specific types of violence against women, others found increased rates of DV against men.

(vi) Pre-existing Violence and Attitudes toward DV

A history of exposure to violence prior to the pandemic, along with personal or community tolerance of violence, significantly heightened the risk of DV during the COVID-19 pandemic.^28,34,37,40,51,57–59^ A history of childhood exposure to family violence further increased vulnerability to DV. Individuals with prior DV experiences were more likely to face repeated abuse, while those without such experiences often encountered violence for the first time. Living in isolation from supportive networks further exacerbated this risk. In societies where DV was normalized or less recognized as abuse, there was a higher tolerance for DV, which led to increased rates during the pandemic.

(vii) Protective Factors

Despite the challenges posed by the pandemic, some studies reported that some protective factors may help reduce the risk of DV.^30,35,50,53^ These included strong community connections, access to social support networks, partner support, higher general self-efficacy, religious beliefs, and adherence to health guidelines. Moreover, the recognition of abusive behaviors was identified as a key protective factor in preventing DV.

The leave-one-out sensitivity analyses (Supplementary Figures 3A–3F) further depict the robustness of the pooled estimates by demonstrating that exclusion of any single study did not substantially alter the overall pooled prevalence. These plots confirm that no individual study disproportionately influenced the results. The results of correlation tests showed no statistically significant multicollinearity issues among independent variables, including age, gender, country of region, and type of DV (P >0.05).

## 4. Discussion

This systematic review and meta-analysis estimated the prevalence of DV in COVID-19 during the COVID-19 pandemic across various demographics, including women, men, the elderly, different regions, including individuals in developed versus developing countries, as well as age categories of those 40 years and younger and over 40. Additionally, it identified key determinants influencing DV rates. Our results indicate that 13% of the population, or 1 in 8 individuals, experienced DV during the pandemic, with psychological violence being the most prevalent type. Notably, the prevalence of DV among women was significantly higher than among men or older adult populations. In this respect, one-fifth of women experienced at least one type of DV during the pandemic. Among them, approximately 30% reported experiencing psychological violence, and 20% reported controlling or threatening behavior. Furthermore, being 40 years and younger of age and living in developing countries were significantly associated with a higher prevalence of DV.

Natural disasters and public health crises can increase the risk of psychosocial harms, including domestic violence. During the COVID-19 pandemic, DV became a critical global concern as lockdowns and movement restrictions forced many victims to remain in close proximity to their abusers for prolonged periods.^12,64^ Survivors who often already face social isolation experienced even greater hardship under quarantine measures. This “double isolation,” coupled with reduced access to formal and informal support systems, enabled abusers to act with fewer barriers and less accountability.^65^

The results of our review study showed that 13% of the population experienced at least one type of DV during the COVID-19 pandemic. This finding is in agreement with the previous study that reported the increasing risk of DV during the COVID-19 pandemic.^13^

Additionally, we found that psychological violence was the most prevalent form of DV in the population, regardless of gender. This may be attributed to higher levels of stressors, such as economic instability and social isolation, which exacerbate tensions within households and reinforce power imbalances. Lockdowns limited victims’ access to support networks, allowing abusers to exert control without scrutiny.^43^ It should be noted that psychological abuse often goes unreported due to its lack of visible signs, which may lead to under-recognition and normalization of these behaviors and make it more common.^66^

Furthermore, our findings indicate that during the COVID-19 pandemic, women are more vulnerable to DV than men or older populations, with an overall prevalence of 18%. In a recently published meta-analysis by Costa et al. (2024), which encompassed 103 studies on intimate partner violence during COVID-19, the pooled prevalence estimate for any type of violence against women was reported to be 21%. This aligns with our findings, which show the significant impact of the pandemic on women’s safety.^67^ This phenomenon may be due to various interrelated factors. The confinement measures forced many women to stay at home with their abusers, exacerbating existing vulnerabilities and limiting their access to support services. Social distancing measures intensified barriers to reporting, such as fear of contamination, aggressive behaviors from abusers, and decreased social support.^68–70^

DV among the elderly is also a critical yet often overlooked issue that poses significant risks to the well-being and safety of older individuals. This form of abuse can manifest in various ways, including physical, emotional, financial, and neglect. Elderly victims frequently face unique vulnerabilities, such as social isolation, dependency on caregivers, and cognitive impairments, which can exacerbate their risk of experiencing abuse. Barriers to reporting, such as fear of retaliation, feelings of shame, and a lack of awareness about their situation, further complicate the issue. The consequences of DV for the elderly are profound, leading to serious physical and mental health issues, as well as social isolation. Addressing this problem requires increased awareness, access to support services, and community involvement to create a safer environment for older adults.^71^

Furthermore, economic stress, job losses, and financial insecurity heightened tensions within households, increasing the likelihood of violence. The pandemic’s restrictions on movement also limited women’s ability to seek help or escape abusive situations.^72,73^

Additionally, in agreement with a previous report,^13^ we found that the prevalence of DV in developing countries was higher than in developed countries. We hypothesized that developed countries usually have greater awareness and better reporting mechanisms, leading to higher documented rates of DV. In contrast, cultural differences also significantly shape perceptions of DV, with some societies normalizing abusive behaviors, cultural stigmas, lack of education, and limited access to support services in many developing countries contributing to underreporting.^74^ Additionally, social norms and gender roles in developed countries may exacerbate DV rates, with issues like substance abuse and economic stressors being more prevalent. The availability of resources and legal protections for victims in developed nations further facilitates reporting, while victims in developing countries often face systemic barriers to seeking help. Moreover, the economic pressures from the COVID-19 pandemic have intensified vulnerabilities in both contexts, but established support systems in developed countries likely lead to higher recognition and documentation of DV cases.^75–77^ Socioeconomic factors, including economic instability and income inequality, also play a critical role in exacerbating DV situations. Lastly, media representation of DV has heightened public awareness, encouraging victims to come forward.^78^ Therefore, understanding the multifaceted nature of DV prevalence in developed countries requires a comprehensive approach that considers these various influencing factors.^79^

However, our findings showed that many factors could influence domestic DV during the COVID-19 pandemic. Economic determinants, such as financial stress and job loss, emerged as significant contributors to rising DV rates, particularly when compounded by sociodemographic factors like age and family size. Relationship dynamics, characterized by behavioral issues, marital status, and conflict with in-laws, also played a critical role in increasing DV risk. The pandemic-related stressors, including prolonged confinement and disrupted daily routines, further exacerbated vulnerabilities. Emotional, mental, and physical health issues were significant predictors, especially among those with a history of violence, which underscores the importance of addressing underlying health conditions. Additionally, the heightened risks faced by individuals with diverse sexual orientations and gender identities call for tailored support strategies. Conversely, the findings of our study highlight the critical role of protective factors, particularly community connections and social support, in mitigating the risk of DV. These elements not only provide individuals with a sense of belonging and security but also serve as essential resources during times of crisis. Strengthening community ties can foster environments where individuals feel safe to seek help and share their experiences, thereby reducing isolation and vulnerability. Furthermore, social support networks can offer emotional and practical assistance, which is crucial for individuals facing DV situations. Therefore, enhancing these protective factors should be a central focus for future interventions and policies aimed at reducing DV. By prioritizing community engagement and support systems, we can create a more resilient society that effectively addresses the complexities of DV, ultimately leading to safer environments for all individuals. Policymakers should prioritize the development of comprehensive strategies that address the unique challenges posed by the pandemic, including increased funding for DV shelters and hotlines, as well as the integration of DV screening protocols within healthcare settings. Healthcare providers play a crucial role in identifying and supporting victims of DV; therefore, training programs should be implemented to equip them with the necessary skills to recognize signs of abuse and respond appropriately. Additionally, telehealth services should be expanded to ensure that individuals can access confidential support and resources safely. By fostering collaboration between healthcare systems, community organizations, and policymakers, we can create a more coordinated response to DV, ultimately improving outcomes for victims during and beyond the pandemic.

The findings from our subgroup analysis indicate that the prevalence of DV varied across different phases of the COVID-19 pandemic, with a notable increase post-lockdown. While DV prevalence remained stable during strict and partial lockdowns, the rise observed post-lockdown may reflect delayed disclosures, long-term socioeconomic stressors, or shifts in reporting behaviors as restrictions eased. The observed increase in psychological violence across all phases suggests persistent emotional distress and strained interpersonal relationships, potentially exacerbated by prolonged uncertainty and financial instability. The decline in sexual violence post-lockdown may be attributed to changes in living arrangements, reduced perpetrator access, or shifts in reporting trends. However, the result of this subgroup analysis should be interpreted with caution due to limited number of studies in post-lockdown situation.

Additionally, it should be noted that the dynamics of DV are complex and multifaceted. A key yet often overlooked aspect of domestic violence is its bidirectional nature, wherein individuals may simultaneously experience victimization and perpetrate abuse.^80,81^ Recognizing this dynamic is essential, particularly in the context of the COVID-19 pandemic, which intensified stressors such as social isolation, economic instability, and mental health strain—conditions that can escalate conflict within households. Despite its importance, the present review was unable to examine the directionality of violence. This limitation stems from the fact that most included studies reported DV solely from the victim’s perspective and rarely collected or disaggregated data on mutual or reciprocal aggression. The absence of such information restricts a more nuanced understanding of relationship dynamics and may underestimate the complexity of DV during public health crises. To address this gap, future research should prioritize dyadic, couple-level, or longitudinal designs capable of capturing mutual patterns of abuse. Such approaches would offer deeper insight into the prevalence, determinants, and consequences of bidirectional violence and support the development of more targeted and comprehensive prevention and intervention strategies.

## 5. Limitations of the review

Our study had some limitations. Substantial heterogeneity was observed across all meta-analyses, reflecting the multifaceted nature of the included studies and the genuine variability in domestic violence prevalence across different populations and settings. This heterogeneity stems from differences in participant demographics (such as age, gender, and socioeconomic status), cultural contexts that influence disclosure of DV, and methodological variations, including study design and data collection tools. The wide range of prevalence estimates such as the markedly higher DV burden among women (18%) compared with men (11%) and older adults (6%), the substantial differences between developing (23%) and developed countries (8%), and the clear gradient by age group indicate that DV risk during the COVID-19 pandemic is strongly influenced by contextual, demographic, and socioeconomic factors. This variability is expected given the inclusion of studies from diverse settings, measurement tools, and pandemic phases. While the high I^2^ values mean that pooled estimates should be interpreted with caution, they do not undermine the reliability of the findings; instead, they highlight important contextual differences across populations. Importantly, our subgroup analyses and meta-regression substantially reduced heterogeneity within categories and identified several key contributors, including country development level, participant age, and gender. These findings provide confidence that while absolute pooled estimates should be interpreted cautiously, the observed patterns higher DV prevalence among women, younger individuals, residents of developing countries, and during the post-lockdown period—remain robust. Together, these results demonstrate that heterogeneity did not obscure the core signals of the review but instead highlighted meaningful differences across contexts that enhance the interpretability and policy relevance of our findings.

Additionally, cultural reporting biases may lead to underreporting in certain contexts while fostering higher visibility in others, complicating the data landscape. While we addressed some of this heterogeneity through subgroup analyses, we recognize the need for a more nuanced exploration of these factors to enhance our understanding of the complexities inherent in the studies included in our meta-analysis.

Our study provides a comprehensive analysis of violence affecting adults, including intimate partner violence and other forms of DV. We excluded child violence from our review, recognizing that it involves distinct risk factors and reporting mechanisms; We also excluded non-English publications which may limit the comprehensiveness of this study and could introduce bias by omitting relevant data from diverse cultural contexts. These omissions may limit the generalizability of our findings regarding DV as a whole. Our findings highlight the need for future research to include a broader range of studies, particularly non-English publications, to reduce bias and improve generalizability. Additionally, grey literature was excluded from this review, as it is often heterogeneous in quality and reporting standards, which could introduce variability and potential bias into the findings. Our search strategy prioritized DV as the primary term to ensure relevance rather than violence alone.

The reliance on point estimates also presents another limitation in our ability to draw meaningful comparisons between pre-pandemic and during or post-pandemic risk differences. This limitation arises from the inherently cross-sectional nature of the data, which captures a snapshot in time rather than longitudinal trends. Consequently, without longitudinal data or baseline measures, we cannot ascertain whether the observed prevalence rates reflect a genuine increase in DV incidents or are merely an artifact of varying reporting practices, societal stressors, or other confounding factors during the pandemic. Despite conducting an extensive literature search, certain unpublished studies and those written in languages other than English were not included. In addition, different tools have been used to assess DV across studies, leading to potential inconsistencies and variations in reported prevalence rates. Furthermore, using self-reported data in many studies may introduce response bias, as participants may underreport or over report their experiences of DV due to stigma or fear of repercussions.

## 6. Conclusion

Our findings indicate that the prevalence of DV during the COVID-19 pandemic was alarmingly high globally, with an overall pooled prevalence of 13%. Psychological violence emerged as the most prevalent subtype. Women, with a risk differential of 15%, and young adults, with a differential of 12%, are recognized as particularly vulnerable populations, especially within the context of developing countries, where the risk differential reaches 14%. By providing a comprehensive assessment of the prevalence of DV across various populations and its determinants, this study contributes valuable insights that can guide future research and inform public health responses to this critical issue.

## Supplemental material

Supplemental material - Global prevalence of domestic violence against adults during COVID-19 and its determinants: A systematic review, meta-analysis, and meta-regression analysis Supplemental material for Global prevalence of domestic violence against adults during COVID-19 and its determinants: A systematic review, meta-analysis, and meta-regression analysis by Razieh Bidhendi-Yarandi, Farhad Nosrati Nejad, Akbar Biglarian, Payam Roshnfekr, Samira Behboudi-Gandevani in Women's Health

Supplemental material - Global prevalence of domestic violence against adults during COVID-19 and its determinants: A systematic review, meta-analysis, and meta-regression analysis Supplemental material for Global prevalence of domestic violence against adults during COVID-19 and its determinants: A systematic review, meta-analysis, and meta-regression analysis by Razieh Bidhendi-Yarandi, Farhad Nosrati Nejad, Akbar Biglarian, Payam Roshnfekr, Samira Behboudi-Gandevani in Women's Health

## Data Availability

Not applicable.*

## Appendix

### Abbreviation

PRISMA-P: Preferred Reporting Items for Systematic Reviews and Meta-analyses protocol.
DV: Domestic violence
WHO: World Health Organization.
