# Supplementary material for: Global prevalence of domestic violence against adults during COVID-19 and its determinants: A systematic review, meta-analysis, and meta-regression analysis
Source: Womens Health (Lond). 2026 Jul 2;22:17455057261465474. doi: 10.1177/17455057261465474 (PMC13329016; doi:10.1177/17455057261465474)
Supplement: Supplemental material - Global prevalence of domestic violence against adults during COVID-19 and its determinants: A systematic review, meta-analysis, and meta-regression analysis [file sj-pdf-1-whe-10.1177_17455057261465474.pdf]

The PRISMA 2020 reporting checklist

For checking that systematic review articles can be understood and used by everyone

	Item Description	Location (or reason for not reporting)
**Title and Abstract**		
1. Title	Identify the report as a systematic review.	#1
2. Abstract	Include all items from the *PRISMA 2020 for Abstracts* checklist.	#1-2
**Introduction**		
3. Rationale	Describe the rationale for the review in the context of existing knowledge.	#3-4
4. Objectives	Provide an explicit statement of the objective(s) or question(s) the review addresses.	#3-4
**Methods**		
5. Eligibility criteria	Specify the inclusion and exclusion criteria for the review and how studies were grouped for the syntheses.	#4-5
6. Information sources	Specify all databases, registers, websites, organisations, reference lists and other sources searched or consulted to identify studies. Specify the date when each source was last searched or consulted.	#5
7. Search	Present the full search strategies for all databases, registers and websites, including any filters and limits used.	#5
8. Selection Process	Specify the methods used to decide whether a study met the inclusion criteria of the review, including how many reviewers screened each record and each report retrieved, whether they worked independently, and, if applicable, details of automation tools used in the process.	#5
9. Data collection process	Specify the methods used to collect data from reports, including how many reviewers collected data from each report, whether they worked independently, any processes for obtaining or confirming data from study investigators, and if applicable, details of automation tools used in the process.	#5-7
10. Data Items		

10a. Outcomes	List and define all outcomes for which data were sought. Specify whether all results that were compatible with each outcome domain in each study were sought (e.g. for all measures, time points, analyses), and if not, the methods used to decide which results to collect.	#5-7
10b. Other Variables	List and define all other variables for which data were sought (e.g. participant and intervention characteristics, funding sources). Describe any assumptions made about any missing or unclear information.	#5-7
11. Risk of bias in individual studies	Specify the methods used to assess risk of bias in the included studies, including details of the tool(s) used, how many reviewers assessed each study and whether they worked independently, and if applicable, details of automation tools used in the process.	#5-7
12. Effect measures	Specify for each outcome the effect measure(s) (e.g. risk ratio, mean difference) used in the synthesis or presentation of results.	#5-7
13. Synthesis Methods		
13a. Deciding which studies were eligible for each synthesis	Describe the processes used to decide which studies were eligible for each synthesis (such as tabulating the study intervention characteristics and comparing against the planned groups for each synthesis described in item 5.	#6-7
13b. Data preparation methods	Describe any methods required to prepare the data for presentation or synthesis, such as handling of missing summary statistics, or data conversions.	#6-7
13c. Methods for tabulating or displaying results	Describe any methods used to tabulate or visually display results of individual studies and syntheses.	#6-7
13d. Synthesis methods	Describe any methods used to synthesize results and provide a rationale for the choice(s). If meta-analysis was performed, describe the model(s), method(s) to identify the presence and extent of statistical heterogeneity, and software package(s) used.	#6-7
13e. Methods for exploring heterogeneity	Describe any methods used to explore possible causes of heterogeneity among study results (e.g. subgroup analysis, meta-regression).	#6-7
13f. Sensitivity analyses	Describe any sensitivity analyses conducted to assess robustness of the synthesized results.	#6-7
14. Reporting bias assessment	Describe any methods used to assess risk of bias due to missing results in a synthesis (arising from	#6-7

	reporting biases).	
15. Certainty assessment	Describe any methods used to assess certainty (or confidence) in the body of evidence for an outcome.	#6-7
**Results**		
16. Study Selection		
16a. Results of the search and selection process	Describe the results of the search and selection process, from the number of records identified in the search to the number of studies included in the review, ideally using a flow diagram.	#7-8
16b. Excluded studies	Cite studies that might appear to meet the inclusion criteria, but which were excluded, and explain why they were excluded.	#7-8
17. Study characteristics	Cite each included study and present its characteristics.	#7-8
18. Risk of bias in studies	Present assessments of risk of bias for each included study.	#8
19. Results of individual studies	For all outcomes, present, for each study: (a) summary statistics for each group (where appropriate) and (b) an effect estimate and its precision (e.g. confidence/credible interval), ideally using structured tables or plots.	#7-8
20. Results of Synthesis		
20a. Summary of studies	For each synthesis, briefly summarise the characteristics and risk of bias among contributing studies.	#8-13
20b. Statistical results	Present results of all statistical syntheses conducted. If meta-analysis was done, present for each the summary estimate and its precision (e.g. confidence/credible interval) and measures of statistical heterogeneity. If comparing groups, describe the direction of the effect.	#8-13
20c. Heterogeneity	Present results of all investigations of possible causes of heterogeneity among study results.	#8-13
20d. Sensitivity analyses	Present results of all sensitivity analyses conducted to assess the robustness of the synthesized results.	#8-13
21. Risk of reporting biases in syntheses	Present assessments of risk of bias due to missing results (arising from reporting biases) for each synthesis assessed.	#8-13
22. Certainty of evidence	Present assessments of certainty (or confidence) in the body of evidence for each outcome assessed.	#8-13
**Discussion**		
23. Discussion		

23a. General interpretation of the results	Provide a general interpretation of the results in the context of other evidence.	#13-17
23b. Limitations of included evidence	Discuss any limitations of the evidence included in the review.	#17-18
23c. Limitations of the review processes	Discuss any limitations of the review processes used.	#17-18
23d. Implications	Discuss implications of the results for practice, policy, and future research.	#18-19
**Other Information**		
24. Registration and Protocol		
24a. Registration	Provide registration information for the review, including register name and registration number, or state that the review was not registered.	#19-20
24b. Protocol	Indicate where the review protocol can be accessed, or state that a protocol was not prepared.	#19-20
24c. Amendments	Describe and explain any amendments to information provided at registration or in the protocol.	#19-20
25. Support	Describe sources of financial or non-financial support for the review, and the role of the funders or sponsors in the review.	#19-20
26. Competing Interests	Declare any competing interests of review authors.	#19-20
27. Availability of data, code, and other materials	Report which of the following are publicly available and where they can be found: template data collection forms; data extracted from included studies; data used for all analyses; analytic code; any other materials used in the review.	#19-20
