# Supplementary material for: Global prevalence of domestic violence against adults during COVID-19 and its determinants: A systematic review, meta-analysis, and meta-regression analysis
Source: Womens Health (Lond). 2026 Jul 2;22:17455057261465474. doi: 10.1177/17455057261465474 (PMC13329016; doi:10.1177/17455057261465474)
Supplement: Supplemental material - Global prevalence of domestic violence against adults during COVID-19 and its determinants: A systematic review, meta-analysis, and meta-regression analysis [file sj-pdf-2-whe-10.1177_17455057261465474.pdf]

Supplementary Table 1. Risk of bias assessment (JBI critical appraisal checklist reporting prevalence data) for assessing the quality of the studies.

	Was the sample frame appropriate to address the target population?	Were study participants sampled in an appropriate way?	Was the sample size adequate?	Were the study subjects and the setting described in detail?	Was the data analysis conducted with sufficient coverage of the identified sample?	Were valid methods used for the identification of the condition?	Was the condition measured in a standard, reliable way for all participants?	Was there appropriate statistical analysis?	Was the response rate adequate, and if not, was the low response rate managed appropriately?
Agero G et al. 2024	Yes	Yes	Yes	Yes	Yes	Yes	Yes	Yes	Yes
Arenas-Arroyo E et al. 2021	Yes	Yes	Yes	Yes	Yes	Yes	Yes	Yes	Yes
Campbell et al. 2023	Yes	Yes	Yes	Yes	Yes	Yes	Yes	Yes	Yes
Chang et al. 2021	Yes	Yes	Yes	Yes	Yes	Yes	Yes	Yes	Yes
Chang et al. 2024	Yes	Yes	Yes	Yes	Yes	Yes	Yes	Yes	Yes
da Costa Siqueira et al. 2023	Yes	Yes	Yes	Yes	Yes	Yes	Yes	Yes	Yes
Demeke et al. 2023	Yes	Yes	Yes	Yes	Yes	Yes	Yes	Yes	Yes
Drieskens et al. 2022	Yes	Yes	Yes	Yes	Yes	Yes	Yes	Yes	Yes
Du et al. 2021	Yes	Yes	Yes	Yes	Yes	Yes	Yes	Yes	Yes
Every-Palmer et al. 2020	Yes	Yes	Yes	Yes	Yes	Yes	Yes	Yes	Yes
Ezenwoko et al. 2023	Yes	Yes	Yes	Yes	Yes	Yes	Yes	Yes	Yes
Fereidooni et al. 2023	Yes	Yes	Yes	Yes	Yes	Yes	Yes	Yes	Yes
Gebrewahd et al. 2020	Yes	Yes	Yes	Yes	Yes	Yes	Yes	Yes	Yes
Gharacheh et al. 2023	Yes	Yes	Yes	Yes	Yes	Yes	Yes	Yes	Yes
Gilchrist et al. 2023	Yes	Yes	Yes	Yes	Yes	Yes	Yes	Yes	Yes
Gleason et al. 2021	Yes	Yes	Yes	Yes	Yes	Yes	Yes	Yes	Yes
Glowacz et al. 2020	Yes	Yes	Yes	Yes	Yes	Yes	Yes	Yes	Yes
Arumugaperumal et al. 2024	Yes	Yes	Yes	Yes	Yes	Yes	Yes	Yes	Yes
Indu et al. 2021	Yes	Yes	Yes	Yes	Yes	Yes	Yes	Yes	Yes
Kliem et al. 2023	Yes	Yes	Yes	Yes	Yes	Yes	Yes	Yes	Yes
Koga et al. 2022	Yes	Yes	Yes	Yes	Yes	Yes	Yes	Yes	Yes
Leite et al. 2023	Yes	Yes	Yes	Yes	Yes	Yes	Yes	Yes	Yes
Miall et al. 2023	Yes	Yes	Yes	Yes	Yes	Yes	Yes	Yes	Yes
Miller et al. 2022	Yes	Yes	Yes	Yes	Yes	Yes	Yes	Yes	Yes
Morgan et al. 2020	Yes	Yes	Yes	Yes	Yes	Yes	Yes	Yes	Yes

O'Hara et al. 2022	Yes	Yes	Yes	Yes	Yes	Yes	Yes	Yes	Yes
Plášilová et al. 2021	Yes	Yes	Yes	Yes	Yes	Yes	Yes	Yes	Yes
Sanz-Barbero et al. 2021	Yes	Yes	Yes	Yes	Yes	Yes	Yes	Yes	Yes
Sheridan-Johnson et al. 2024	Yes	Yes	Yes	Yes	Yes	Yes	Yes	Yes	Yes
Shewangzaw Engda et al. 2022	Yes	Yes	Yes	Yes	Yes	Yes	Yes	Yes	Yes
Shitu et al. 2021	Yes	Yes	Yes	Yes	Yes	Yes	Yes	Yes	Yes
Son et al. 2022	Yes	Yes	Yes	Yes	Yes	Yes	Yes	Yes	Yes
Tadesse et al. 2022	Yes	Yes	Yes	Yes	Yes	Yes	Yes	Yes	Yes
Yan et al. 2022	Yes	Yes	Yes	Yes	Yes	Yes	Yes	Yes	Yes
Yan et al. 2022	Yes	Yes	Yes	Yes	Yes	Yes	Yes	Yes	Yes
Yang et al. 2024	Yes	Yes	Yes	Yes	Yes	Yes	Yes	Yes	Yes
Yari et al. 2021	Yes	Yes	Yes	Yes	Yes	Yes	Yes	Yes	Yes

Supplementary figures 1A-C: Forest plots

A

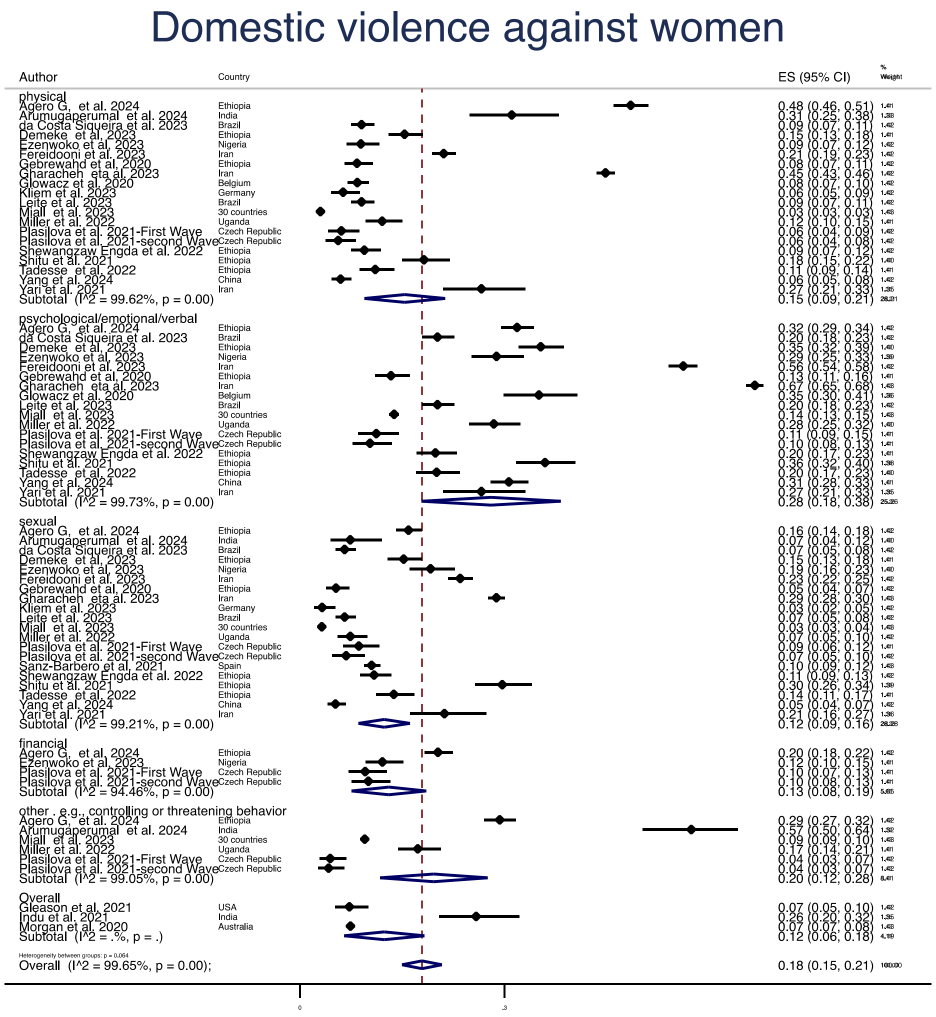
Domestic violence against women
Author
Country
ES (95% CI)
% Weight
physical
Agero G, et al. 2024 Ethiopia 0.48 (0.46, 0.51)
Arumugaperumal et al. 2024 India 0.31 (0.25, 0.38)
da Costa Siqueira et al. 2023 Brazil 0.09 (0.07, 0.11)
Demeke et al. 2023 Ethiopia 0.15 (0.13, 0.18)
Ezenwoko et al. 2023 Nigeria 0.09 (0.07, 0.12)
Fereidooni et al. 2023 Iran 0.21 (0.19, 0.23)
Gebrewahd et al. 2020 Ethiopia 0.08 (0.07, 0.11)
Gharacheh eta al. 2023 Iran 0.45 (0.43, 0.46)
Glowacz et al. 2020 Belgium 0.08 (0.07, 0.10)
Kliem et al. 2023 Germany 0.06 (0.05, 0.09)
Leite et al. 2023 Brazil 0.09 (0.07, 0.11)
Miall et al. 2023 30 countries 0.03 (0.03, 0.03)
Miller et al. 2022 Uganda 0.12 (0.10, 0.15)
Plasilova et al. 2021-First Wave Czech Republic 0.06 (0.04, 0.09)
Plasilova et al. 2021-second Wave Czech Republic 0.06 (0.04, 0.08)
Shewangzaw Engda et al. 2022 Ethiopia 0.09 (0.07, 0.12)
Shitu et al. 2021 Ethiopia 0.18 (0.15, 0.22)
Tadesse et al. 2022 Ethiopia 0.11 (0.09, 0.14)
Yang et al. 2024 China 0.06 (0.05, 0.08)
Yari et al. 2021 Iran 0.27 (0.21, 0.33)
Subtotal (I^2 = 99.62%, p = 0.00) 0.15 (0.09, 0.21)
psychological/emotional/verbal
Agero G, et al. 2024 Ethiopia 0.32 (0.29, 0.34)
da Costa Siqueira et al. 2023 Brazil 0.20 (0.18, 0.23)
Demeke et al. 2023 Ethiopia 0.35 (0.32, 0.39)
Ezenwoko et al. 2023 Nigeria 0.29 (0.25, 0.33)
Fereidooni et al. 2023 Iran 0.56 (0.54, 0.58)
Gebrewahd et al. 2020 Ethiopia 0.13 (0.11, 0.16)
Gharacheh eta al. 2023 Iran 0.67 (0.65, 0.68)
Glowacz et al. 2020 Belgium 0.35 (0.30, 0.41)
Leite et al. 2023 Brazil 0.20 (0.18, 0.23)
Miall et al. 2023 30 countries 0.14 (0.13, 0.15)
Miller et al. 2022 Uganda 0.28 (0.25, 0.32)
Plasilova et al. 2021-First Wave Czech Republic 0.11 (0.09, 0.15)
Plasilova et al. 2021-second Wave Czech Republic 0.10 (0.08, 0.13)
Shewangzaw Engda et al. 2022 Ethiopia 0.20 (0.17, 0.23)
Shitu et al. 2021 Ethiopia 0.36 (0.32, 0.40)
Tadesse et al. 2022 Ethiopia 0.20 (0.17, 0.23)
Yang et al. 2024 China 0.31 (0.28, 0.33)
Yari et al. 2021 Iran 0.27 (0.21, 0.33)
Subtotal (I^2 = 99.73%, p = 0.00) 0.28 (0.18, 0.38)
sexual
Agero G, et al. 2024 Ethiopia 0.16 (0.14, 0.18)
Arumugaperumal et al. 2024 India 0.07 (0.04, 0.12)
da Costa Siqueira et al. 2023 Brazil 0.07 (0.05, 0.08)
Demeke et al. 2023 Ethiopia 0.15 (0.13, 0.18)
Ezenwoko et al. 2023 Nigeria 0.19 (0.16, 0.23)
Fereidooni et al. 2023 Iran 0.23 (0.22, 0.25)
Gebrewahd et al. 2020 Ethiopia 0.05 (0.04, 0.07)
Gharacheh eta al. 2023 Iran 0.29 (0.28, 0.30)
Kliem et al. 2023 Germany 0.03 (0.02, 0.05)
Leite et al. 2023 Brazil 0.07 (0.05, 0.08)
Miall et al. 2023 30 countries 0.03 (0.03, 0.04)
Miller et al. 2022 Uganda 0.07 (0.05, 0.10)
Plasilova et al. 2021-First Wave Czech Republic 0.09 (0.06, 0.12)
Plasilova et al. 2021-second Wave Czech Republic 0.07 (0.05, 0.10)
Sanz-Barbero et al. 2021 Spain 0.10 (0.09, 0.12)
Shewangzaw Engda et al. 2022 Ethiopia 0.11 (0.09, 0.13)
Shitu et al. 2021 Ethiopia 0.30 (0.26, 0.34)
Tadesse et al. 2022 Ethiopia 0.14 (0.11, 0.17)
Yang et al. 2024 China 0.05 (0.04, 0.07)
Yari et al. 2021 Iran 0.21 (0.16, 0.27)
Subtotal (I^2 = 99.21%, p = 0.00) 0.12 (0.09, 0.16)
financial
Agero G, et al. 2024 Ethiopia 0.20 (0.18, 0.22)
Ezenwoko et al. 2023 Nigeria 0.12 (0.10, 0.15)
Plasilova et al. 2021-First Wave Czech Republic 0.10 (0.07, 0.13)
Plasilova et al. 2021-second Wave Czech Republic 0.10 (0.08, 0.13)
Subtotal (I^2 = 94.46%, p = 0.00) 0.13 (0.08, 0.19)
other . e.g., controlling or threatening behavior
Agero G, et al. 2024 Ethiopia 0.29 (0.27, 0.32)
Arumugaperumal et al. 2024 India 0.57 (0.50, 0.64)
Miall et al. 2023 30 countries 0.09 (0.09, 0.10)
Miller et al. 2022 Uganda 0.17 (0.14, 0.21)
Plasilova et al. 2021-First Wave Czech Republic 0.04 (0.03, 0.07)
Plasilova et al. 2021-second Wave Czech Republic 0.04 (0.03, 0.07)
Subtotal (I^2 = 99.05%, p = 0.00) 0.20 (0.12, 0.28)
Overall
Gleason et al. 2021 USA 0.07 (0.05, 0.10)
Indu et al. 2021 India 0.26 (0.20, 0.32)
Morgan et al. 2020 Australia 0.07 (0.07, 0.08)
Subtotal (I^2 = .%, p = .) 0.12 (0.06, 0.18)
Heterogeneity between groups: p = 0.064
Overall (I^2 = 99.65%, p = 0.00); 0.18 (0.15, 0.21)
0
.3

B

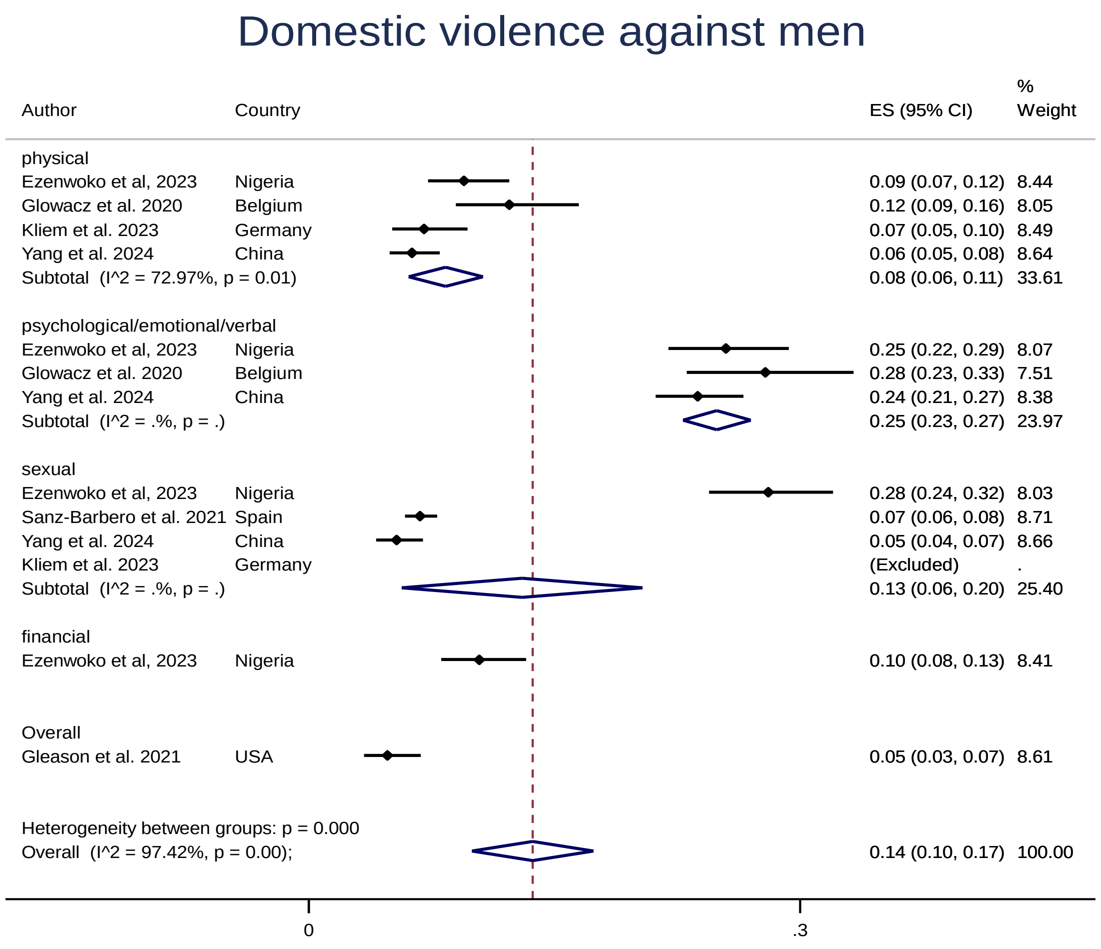
Domestic violence against men
Author
Country
ES (95% CI)
% Weight
physical
Ezenwoko et al, 2023 Nigeria 0.09 (0.07, 0.12) 8.44
Glowacz et al. 2020 Belgium 0.12 (0.09, 0.16) 8.05
Kliem et al. 2023 Germany 0.07 (0.05, 0.10) 8.49
Yang et al. 2024 China 0.06 (0.05, 0.08) 8.64
Subtotal (I^2 = 72.97%, p = 0.01) 0.08 (0.06, 0.11) 33.61
psychological/emotional/verbal
Ezenwoko et al, 2023 Nigeria 0.25 (0.22, 0.29) 8.07
Glowacz et al. 2020 Belgium 0.28 (0.23, 0.33) 7.51
Yang et al. 2024 China 0.24 (0.21, 0.27) 8.38
Subtotal (I^2 = .%, p = .) 0.25 (0.23, 0.27) 23.97
sexual
Ezenwoko et al, 2023 Nigeria 0.28 (0.24, 0.32) 8.03
Sanz-Barbero et al. 2021 Spain 0.07 (0.06, 0.08) 8.71
Yang et al. 2024 China 0.05 (0.04, 0.07) 8.66
Kliem et al. 2023 Germany (Excluded) .
Subtotal (I^2 = .%, p = .) 0.13 (0.06, 0.20) 25.40
financial
Ezenwoko et al, 2023 Nigeria 0.10 (0.08, 0.13) 8.41
Overall
Gleason et al. 2021 USA 0.05 (0.03, 0.07) 8.61
Heterogeneity between groups: p = 0.000
Overall (I^2 = 97.42%, p = 0.00); 0.14 (0.10, 0.17) 100.00
0
.3

C

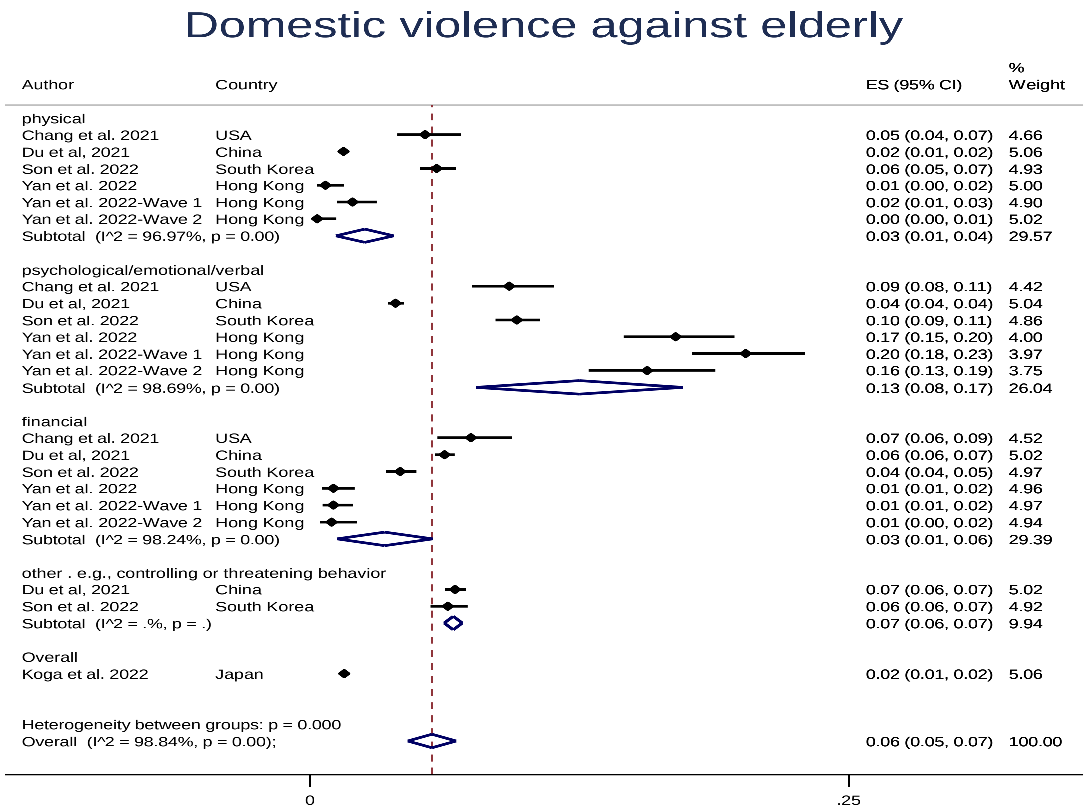
Domestic violence against elderly
Author
Country
ES (95% CI)
% Weight
physical
Chang et al. 2021 USA 0.05 (0.04, 0.07) 4.66
Du et al, 2021 China 0.02 (0.01, 0.02) 5.06
Son et al. 2022 South Korea 0.06 (0.05, 0.07) 4.93
Yan et al. 2022 Hong Kong 0.01 (0.00, 0.02) 5.00
Yan et al. 2022-Wave 1 Hong Kong 0.02 (0.01, 0.03) 4.90
Yan et al. 2022-Wave 2 Hong Kong 0.00 (0.00, 0.01) 5.02
Subtotal (I^2 = 96.97%, p = 0.00) 0.03 (0.01, 0.04) 29.57
psychological/emotional/verbal
Chang et al. 2021 USA 0.09 (0.08, 0.11) 4.42
Du et al, 2021 China 0.04 (0.04, 0.04) 5.04
Son et al. 2022 South Korea 0.10 (0.09, 0.11) 4.86
Yan et al. 2022 Hong Kong 0.17 (0.15, 0.20) 4.00
Yan et al. 2022-Wave 1 Hong Kong 0.20 (0.18, 0.23) 3.97
Yan et al. 2022-Wave 2 Hong Kong 0.16 (0.13, 0.19) 3.75
Subtotal (I^2 = 98.69%, p = 0.00) 0.13 (0.08, 0.17) 26.04
financial
Chang et al. 2021 USA 0.07 (0.06, 0.09) 4.52
Du et al, 2021 China 0.06 (0.06, 0.07) 5.02
Son et al. 2022 South Korea 0.04 (0.04, 0.05) 4.97
Yan et al. 2022 Hong Kong 0.01 (0.01, 0.02) 4.96
Yan et al. 2022-Wave 1 Hong Kong 0.01 (0.01, 0.02) 4.97
Yan et al. 2022-Wave 2 Hong Kong 0.01 (0.00, 0.02) 4.94
Subtotal (I^2 = 98.24%, p = 0.00) 0.03 (0.01, 0.06) 29.39
other . e.g., controlling or threatening behavior
Du et al, 2021 China 0.07 (0.06, 0.07) 5.02
Son et al. 2022 South Korea 0.06 (0.06, 0.07) 4.92
Subtotal (I^2 = .%, p = .) 0.07 (0.06, 0.07) 9.94
Overall
Koga et al. 2022 Japan 0.02 (0.01, 0.02) 5.06
Heterogeneity between groups: p = 0.000
Overall (I^2 = 98.84%, p = 0.00); 0.06 (0.05, 0.07) 100.00
0
.25

Supplementary figures 2A-O: funnel plots, (p=P-value for Publication bias test)

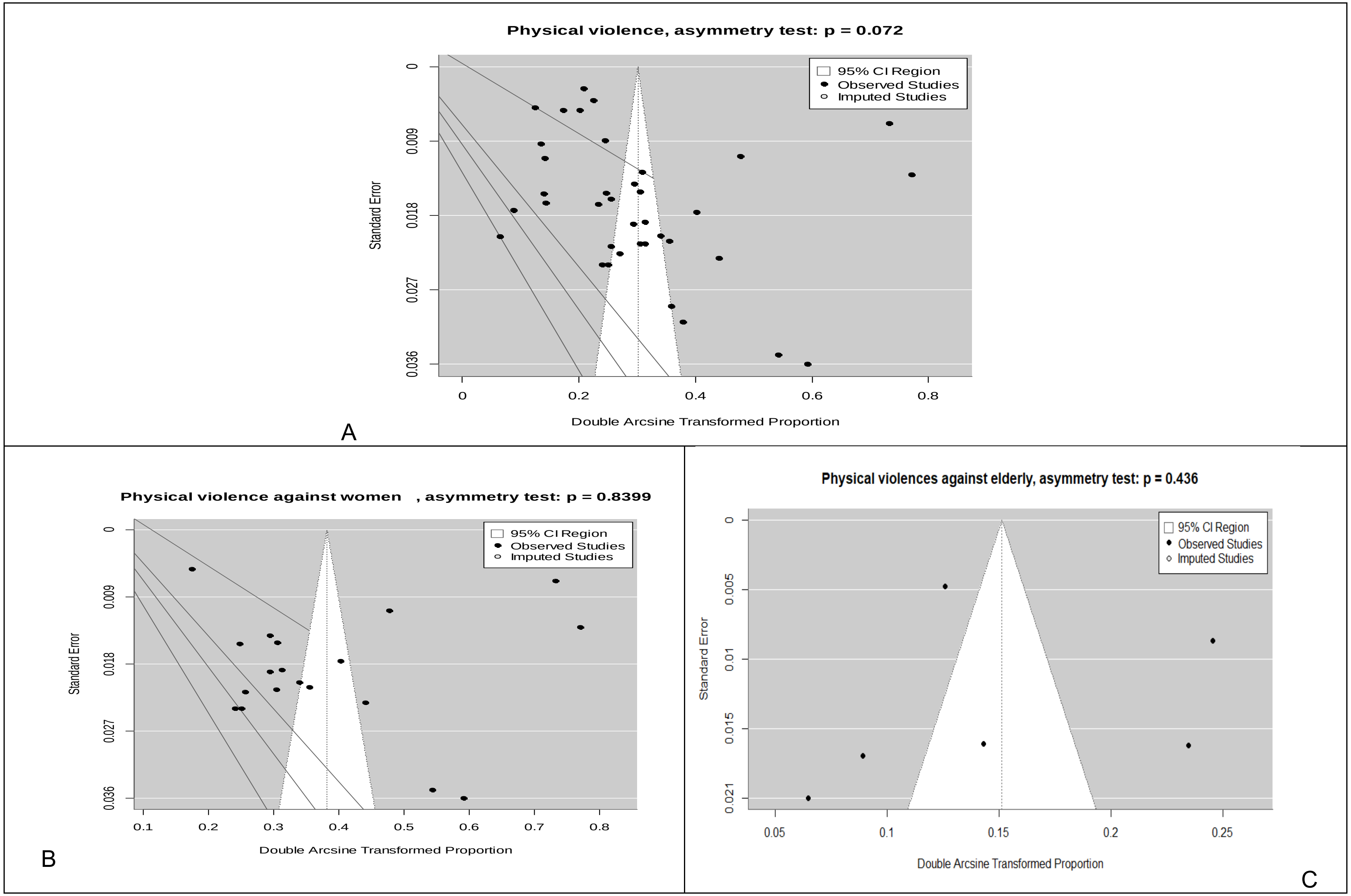

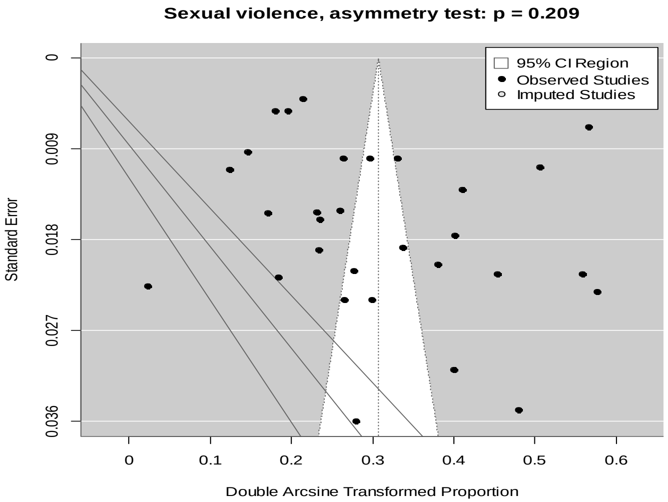
Sexual violence, asymmetry test: p = 0.209
95% CI Region
Observed Studies
Imputed Studies
Standard Error
0
0.009
0.018
0.027
0.036
0
0.1
0.2
0.3
0.4
0.5
0.6
Double Arcsine Transformed Proportion

D

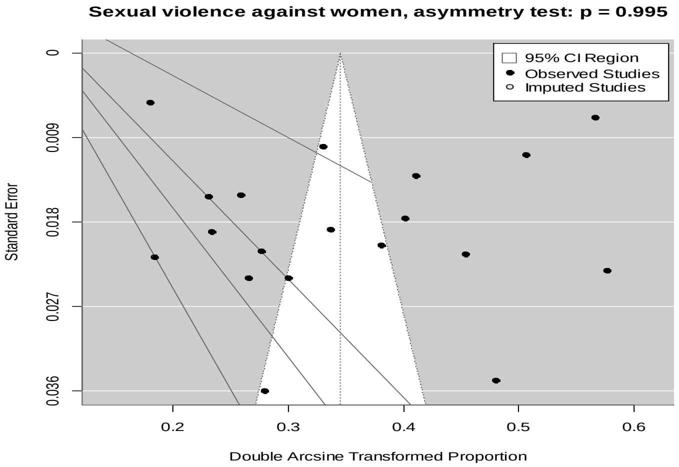
Sexual violence against women, asymmetry test: p = 0.995
95% CI Region
Observed Studies
Imputed Studies
Standard Error
0
0.009
0.018
0.027
0.036
0.2
0.3
0.4
0.5
0.6
Double Arcsine Transformed Proportion

E

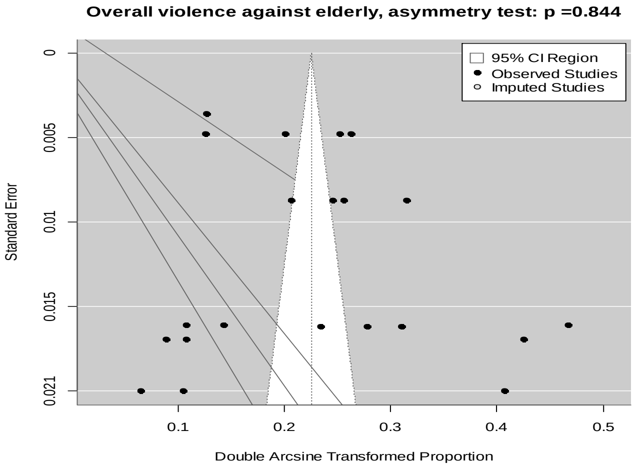
Overall violence against elderly, asymmetry test: p =0.844
95% CI Region
Observed Studies
Imputed Studies
Standard Error
0
0.005
0.01
0.015
0.021
0.1
0.2
0.3
0.4
0.5
Double Arcsine Transformed Proportion

F

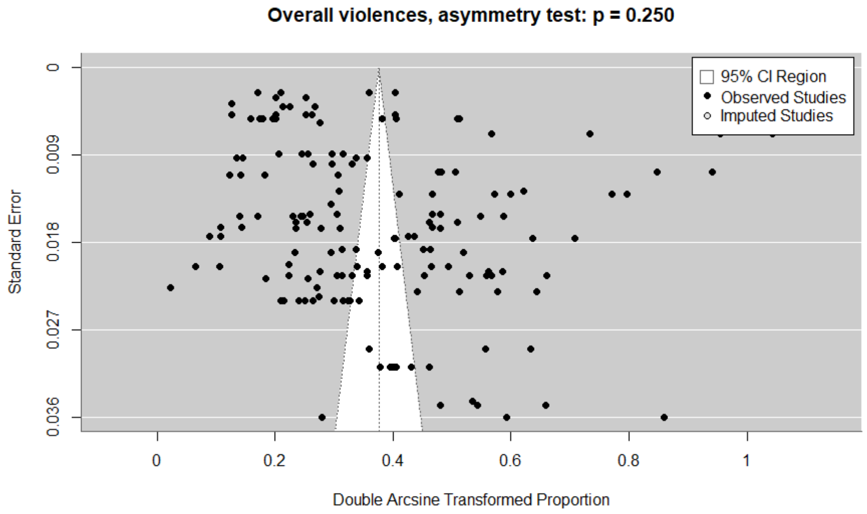
Overall violences, asymmetry test: p = 0.250
95% CI Region
Observed Studies
Imputed Studies
Standard Error
0
0.009
0.018
0.027
0.036
0
0.2
0.4
0.6
0.8
1
Double Arcsine Transformed Proportion

G

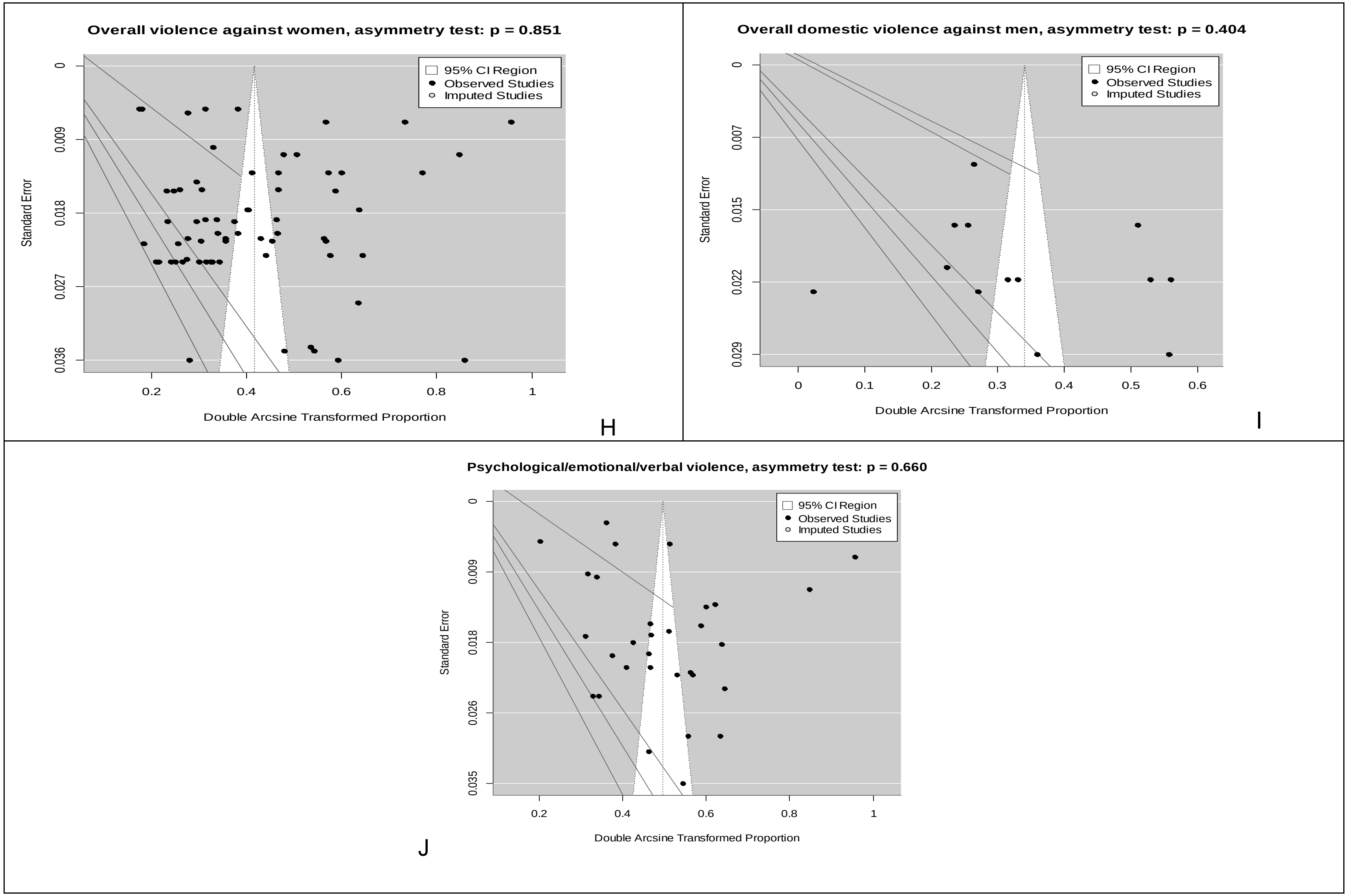
Overall violence against women, asymmetry test: p = 0.851
95% CI Region
Observed Studies
Imputed Studies
Standard Error
0
0.009
0.018
0.027
0.036
0.2
0.4
0.6
0.8
1
Double Arcsine Transformed Proportion
H
Overall domestic violence against men, asymmetry test: p = 0.404
95% CI Region
Observed Studies
Imputed Studies
Standard Error
0
0.007
0.015
0.022
0.029
0
0.1
0.2
0.3
0.4
0.5
0.6
Double Arcsine Transformed Proportion
I
Psychological/emotional/verbal violence, asymmetry test: p = 0.660
95% CI Region
Observed Studies
Imputed Studies
Standard Error
0
0.009
0.018
0.026
0.035
0.2
0.4
0.6
0.8
1
Double Arcsine Transformed Proportion
J

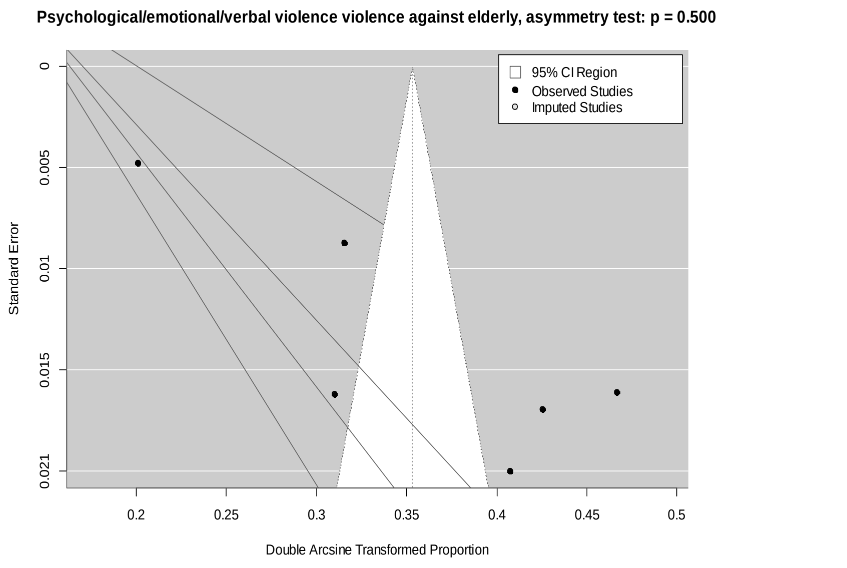
Psychological/emotional/verbal violence violence against elderly, asymmetry test: p = 0.500
95% CI Region
Observed Studies
Imputed Studies
Standard Error
0
0.005
0.01
0.015
0.021
0.2
0.25
0.3
0.35
0.4
0.45
0.5
Double Arcsine Transformed Proportion
K

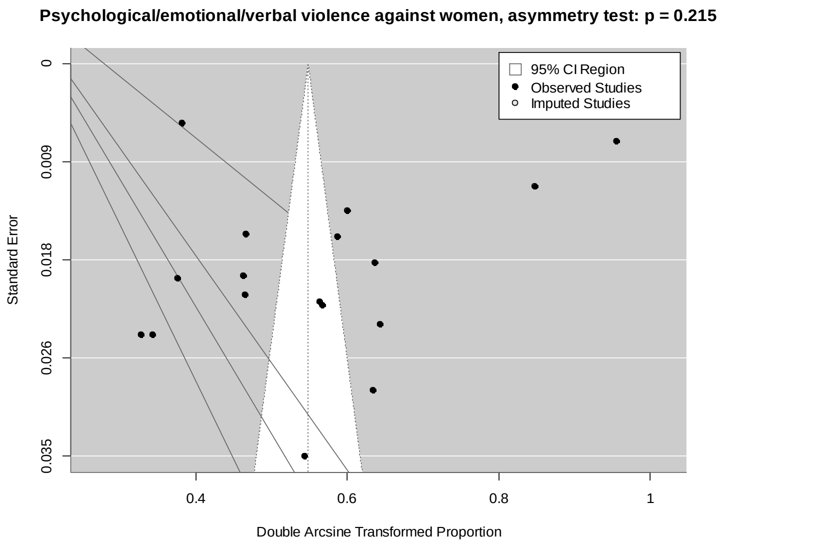
Psychological/emotional/verbal violence against women, asymmetry test: p = 0.215
95% CI Region
Observed Studies
Imputed Studies
Standard Error
0
0.009
0.018
0.026
0.035
0.4
0.6
0.8
1
Double Arcsine Transformed Proportion
L

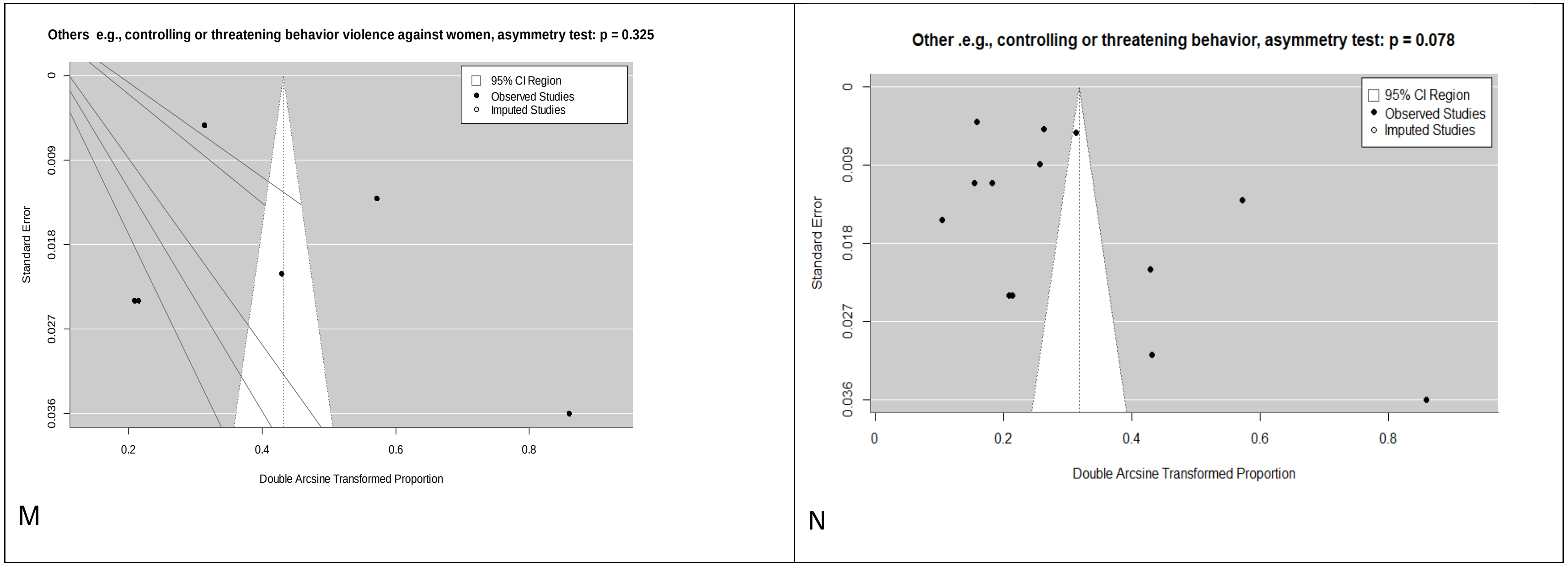
Others e.g., controlling or threatening behavior violence against women, asymmetry test: p = 0.325
95% CI Region
Observed Studies
Imputed Studies
Standard Error
0
0.009
0.018
0.027
0.036
0.2
0.4
0.6
0.8
Double Arcsine Transformed Proportion
M
Other .e.g., controlling or threatening behavior, asymmetry test: p = 0.078
95% CI Region
Observed Studies
Imputed Studies
Standard Error
0
0.009
0.018
0.027
0.036
0
0.2
0.4
0.6
0.8
Double Arcsine Transformed Proportion
N

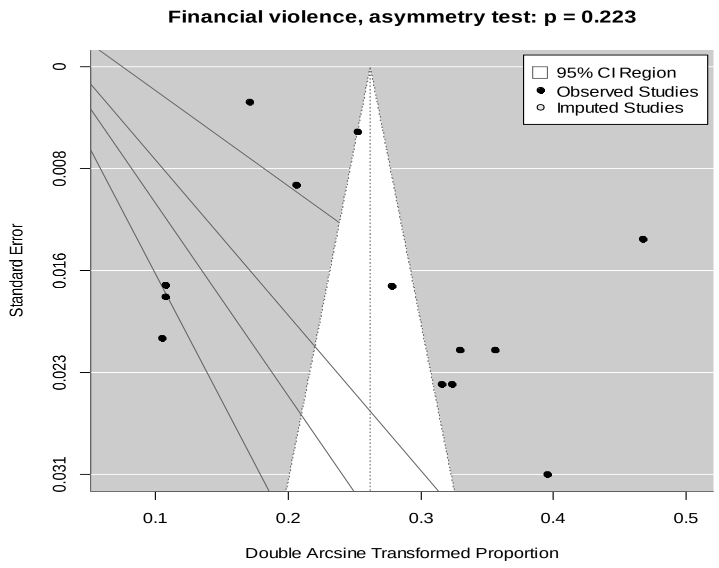

Financial violence, asymmetry test: p = 0.223
95% CI Region
Observed Studies
Imputed Studies
Standard Error
0
0.008
0.016
0.023
0.031
0.1
0.2
0.3
0.4
0.5
Double Arcsine Transformed Proportion

O

Supplementary figures 3A-F:Sensitivity analysis Leave-one-out plots

A:Physical

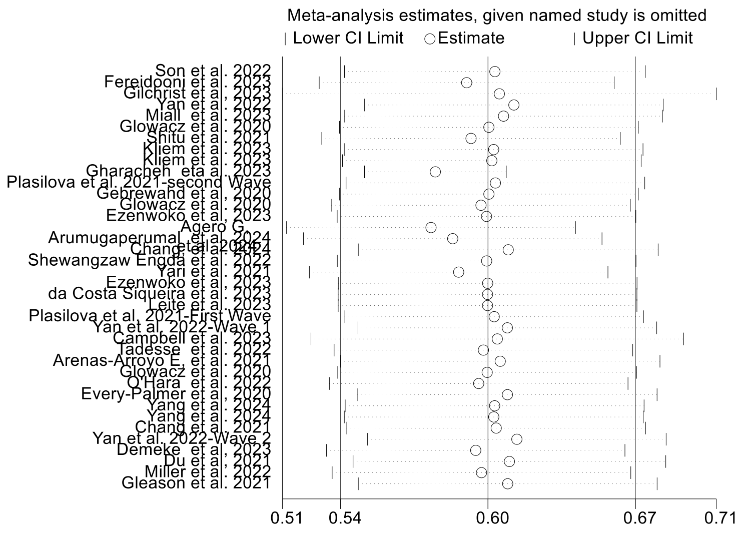

B: Psychological /Emotional/Verbal

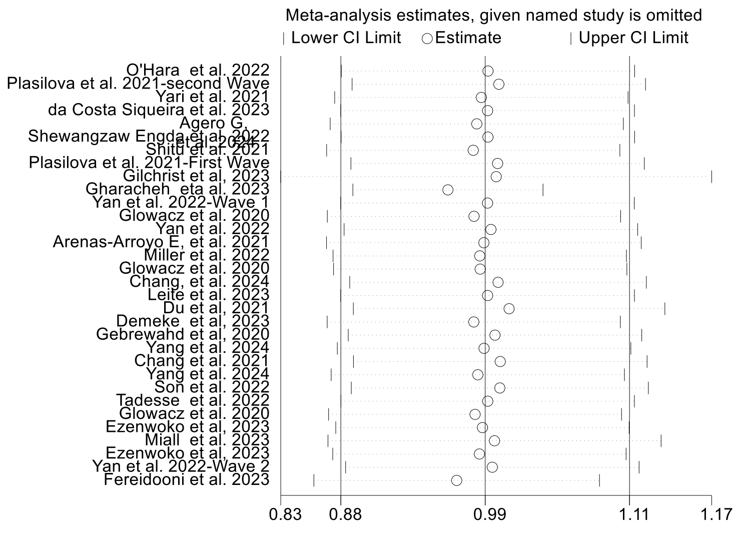

C:Sexual

D:Financial

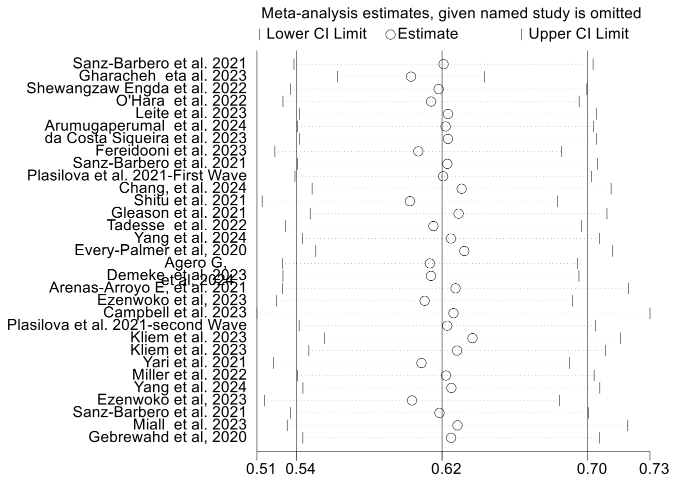

E:Others

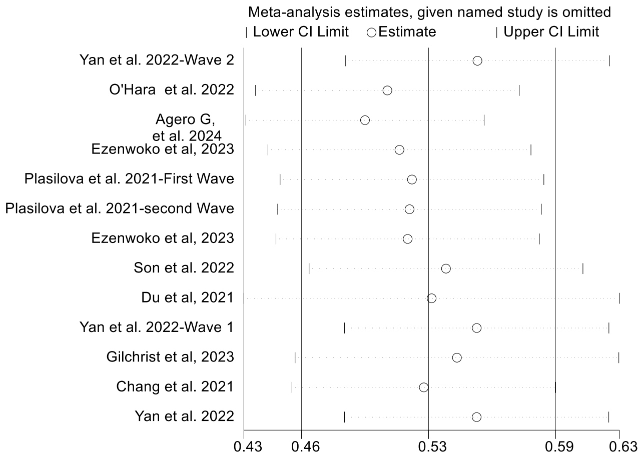

F:Overall

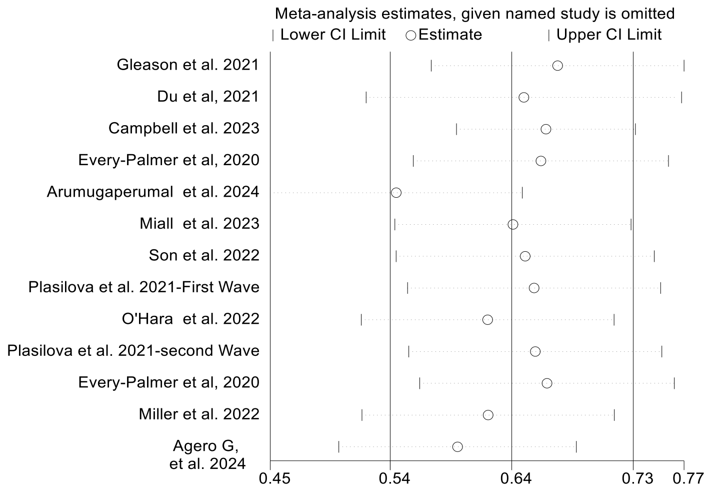
Meta-analysis estimates, given named study is omitted
Lower CI Limit
Estimate
Upper CI Limit
Gleason et al. 2021
Du et al, 2021
Campbell et al. 2023
Every-Palmer et al, 2020
Arumugaperumal et al. 2024
Miall et al. 2023
Son et al. 2022
Plasilova et al. 2021-First Wave
O'Hara et al. 2022
Plasilova et al. 2021-second Wave
Every-Palmer et al, 2020
Miller et al. 2022
Agero G,
et al. 2024
0.45
0.54
0.64
0.73
0.77

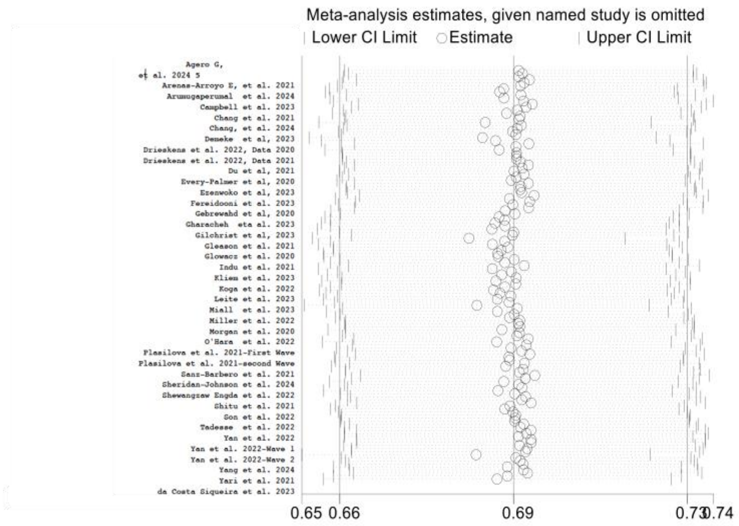
Meta-analysis estimates, given named study is omitted
Lower CI Limit
Estimate
Upper CI Limit
Agero G,
et al. 2024 5
Arenas-Arroyo E, et al. 2021
Arumugaperumal et al. 2024
Campbell et al. 2023
Chang et al. 2021
Chang, et al. 2024
Demeke et al, 2023
Drieskens et al. 2022, Data 2020
Drieskens et al. 2022, Data 2021
Du et al, 2021
Every-Palmer et al, 2020
Ezenwoko et al, 2023
Fereidooni et al. 2023
Gebrewahd et al, 2020
Gharacheh eta al. 2023
Gilchrist et al, 2023
Gleason et al. 2021
Glowacz et al. 2020
Indu et al. 2021
Kliem et al. 2023
Koga et al. 2022
Leite et al. 2023
Miall et al. 2023
Miller et al. 2022
Morgan et al. 2020
O'Hara et al. 2022
Plasilova et al. 2021-First Wave
Plasilova et al. 2021-second Wave
Sanz-Barbero et al. 2021
Sheridan-Johnson et al. 2024
Shewangzaw Engda et al. 2022
Shitu et al. 2021
Son et al. 2022
Tadesse et al. 2022
Yan et al. 2022
Yan et al. 2022-Wave 1
Yan et al. 2022-Wave 2
Yang et al. 2024
Yari et al. 2021
da Costa Siqueira et al. 2023
0.65
0.66
0.69
0.73
0.74

Table 2. Summary of determinant categories, definitions, and examples

Determinant Category	Definition	Examples	Notes/Links to Subgroup Findings
**Economic Stressors**	Financial or material hardship affecting household stability	Job loss, income reduction, inability to meet basic needs	Higher DV prevalence in developing regions; financial violence patterns align with this group
**Lockdown-Related Restrictions**	Changes imposed by pandemic control policies that alter daily life and mobility	Home confinement, limited access to support services, school closures	Related to increased psychological DV during strict/partial lockdown phases
**Psychosocial Stressors**	Individual or interpersonal psychological changes heightened during the pandemic	Depression, anxiety, substance use, relationship conflict	Consistent with higher prevalence among younger adults, women
**Contextual/Demographic Factors**	Pre-existing characteristics influencing vulnerability	Gender, age, living arrangements, prior DV history	Explains differences across participant groups (women, men, elderly)
